# The epidemiology of psoriatic arthritis in the UK: a health intelligence analysis of UK Primary Care Electronic Health Records 1991–2020

**DOI:** 10.1093/rheumatology/kead586

**Published:** 2023-11-02

**Authors:** Katie L Druce, Belay Birlie Yimer, Jennifer Humphreys, Lucy N Njuki, Darryl Bourke, Michael Li, Benjamin Ellis, Yuanyuan Zhang, Ramiro Bravo, Kimme L Hyrich, Suzanne M M Verstappen, William G Dixon, John McBeth

**Affiliations:** Centre for Epidemiology Versus Arthritis, Division of Musculoskeletal and Dermatological Sciences, Faculty of Biology Medicine and Health, The University of Manchester, Manchester Academic Health Science Centre, Manchester, UK; Centre for Epidemiology Versus Arthritis, Division of Musculoskeletal and Dermatological Sciences, Faculty of Biology Medicine and Health, The University of Manchester, Manchester Academic Health Science Centre, Manchester, UK; Centre for Epidemiology Versus Arthritis, Division of Musculoskeletal and Dermatological Sciences, Faculty of Biology Medicine and Health, The University of Manchester, Manchester Academic Health Science Centre, Manchester, UK; NIHR Manchester Biomedical Research Centre, Manchester University NHS Foundation Trust, Manchester, UK; Centre for Epidemiology Versus Arthritis, Division of Musculoskeletal and Dermatological Sciences, Faculty of Biology Medicine and Health, The University of Manchester, Manchester Academic Health Science Centre, Manchester, UK; Centre for Epidemiology Versus Arthritis, Division of Musculoskeletal and Dermatological Sciences, Faculty of Biology Medicine and Health, The University of Manchester, Manchester Academic Health Science Centre, Manchester, UK; Versus Arthritis, London, UK; Versus Arthritis, London, UK; Imperial College Healthcare NHS Trust, London, UK; Centre for Epidemiology Versus Arthritis, Division of Musculoskeletal and Dermatological Sciences, Faculty of Biology Medicine and Health, The University of Manchester, Manchester Academic Health Science Centre, Manchester, UK; Centre for Epidemiology Versus Arthritis, Division of Musculoskeletal and Dermatological Sciences, Faculty of Biology Medicine and Health, The University of Manchester, Manchester Academic Health Science Centre, Manchester, UK; Centre for Epidemiology Versus Arthritis, Division of Musculoskeletal and Dermatological Sciences, Faculty of Biology Medicine and Health, The University of Manchester, Manchester Academic Health Science Centre, Manchester, UK; NIHR Manchester Biomedical Research Centre, Manchester University NHS Foundation Trust, Manchester, UK; Centre for Epidemiology Versus Arthritis, Division of Musculoskeletal and Dermatological Sciences, Faculty of Biology Medicine and Health, The University of Manchester, Manchester Academic Health Science Centre, Manchester, UK; NIHR Manchester Biomedical Research Centre, Manchester University NHS Foundation Trust, Manchester, UK; Centre for Epidemiology Versus Arthritis, Division of Musculoskeletal and Dermatological Sciences, Faculty of Biology Medicine and Health, The University of Manchester, Manchester Academic Health Science Centre, Manchester, UK; NIHR Manchester Biomedical Research Centre, Manchester University NHS Foundation Trust, Manchester, UK; Centre for Epidemiology Versus Arthritis, Division of Musculoskeletal and Dermatological Sciences, Faculty of Biology Medicine and Health, The University of Manchester, Manchester Academic Health Science Centre, Manchester, UK; NIHR Manchester Biomedical Research Centre, Manchester University NHS Foundation Trust, Manchester, UK

**Keywords:** psoriatic arthritis, epidemiology, United Kingdom, Bayesian, misclassification

## Abstract

**Objectives:**

Epidemiological estimates of psoriatic arthritis (PsA) underpin the provision of healthcare, research, and the work of government, charities and patient organizations. Methodological problems impacting prior estimates include small sample sizes, incomplete case ascertainment, and representativeness. We developed a statistical modelling strategy to provide contemporary prevalence and incidence estimates of PsA from 1991 to 2020 in the UK.

**Methods:**

Data from Clinical Practice Research Datalink (CPRD) were used to identify cases of PsA between 1^st^ January 1991 and 31^st^ December 2020. To optimize ascertainment, we identified cases of *Definite PsA (*≥1 Read code for PsA) and *Probable PsA* (satisfied a bespoke algorithm). Standardized annual rates were calculated using Bayesian multilevel regression with post-stratification to account for systematic differences between CPRD data and the UK population, based on age, sex, socioeconomic status and region of residence.

**Results:**

A total of 26 293 recorded PsA cases (all definitions) were identified within the study window (77.9% Definite PsA). Between 1991 and 2020 the standardized prevalence of PsA increased twelve-fold from 0.03–0.37. The standardized incidence of PsA per 100 000 person years increased from 8.97 in 1991–15.08 in 2020, an almost 2-fold increase. Over time, rates were similar between the sexes, and across socioeconomic status. Rates were strongly associated with age, and consistently highest in Northern Ireland.

**Conclusion:**

The prevalence and incidence of PsA recorded in primary care has increased over the last three decades. The modelling strategy presented can be used to provide contemporary prevalence estimates for musculoskeletal disease using routinely collected primary care data.

Rheumatology key messages

- Epidemiological estimates of psoriatic arthritis (PsA) are impacted by methodological challenges that must be overcome.
- The incidence and prevalence of recorded PsA in the UK has increased in the past 30 years.
- Our modelling strategy can provide contemporary prevalence and incidence estimates using routinely collected primary care data.

## Introduction

PsA, a progressive and destructive inflammatory arthritis [1], is closely associated with psoriasis (PsO), an immune-mediated inflammatory skin disease. PsA impacts the quality of life, functional ability, and mortality of affected individuals [1]. Contemporary estimates of PsA prevalence and incidence inform health-care research, service delivery, and resource allocation, both nationally and locally. Estimates would also interest charities and patient organizations who provide support to persons living with PsA, as well as local and national government bodies.

Global estimates of PsA prevalence range from 0.3% to 1% [2, 3], a 3-fold difference, and of PsA incidence range from 3.4 to 8.0 per 100 000 people [2], a 2-fold difference. Evidence from a meta-analysis of 28 studies has provided a more conservative prevalence estimate, of 0.13% [4], with an incidence of 83 per 100 000 person-years, but the data were notably described as having ‘dramatically high’ heterogeneity. Undoubtedly, variation in genetic, environmental and other exposures contribute to the wide range in estimates, but so too will methodological limitations such as differences in sample size, population representativeness, and case ascertainment [4, 5]. Estimates are also impacted by the criteria used, especially if the criteria require serology (e.g. the patient is not RF positive) or assessment by a rheumatologist or medication prescriptions, and the length of time under study [4, 6, 7].

To overcome these issues, UK studies of the epidemiology of PsA have identified cases based on a record of a PsA diagnostic code in primary care health records. However, the studies’ authors have identified other challenges, such as records being impacted by delayed diagnosis (psoriasis and arthritis onset may not co-occur), misclassification (if individual diagnoses are not superseded by a confirmed diagnosis of PsA), or a missing diagnosis if (a) patients do not present to relevant health professionals following referrals, or (b) if medical records are not transferred between care providers, or are not updated with information from secondary care [2, 8, 9].

Identifying patients with ‘probable PsA’ and utilizing models with adjustments for misclassification could improve the accuracy of estimates. However, currently available approaches for misclassification adjustment require unbiased estimates of diagnostic accuracy. In their absence, Bayesian modelling with appropriate prior information reflecting uncertainty in the sensitivity and specificity of case identification can be used.

This study sought to report the annual UK prevalence and incidence of PsA in adults ≥18 years from 1991 to 2020. The specific objectives were to (1) develop a statistical modelling strategy for overcoming identified methodological challenges, (2) provide contemporary epidemiological estimates, (3) examine temporal changes from 1991 to 2020 and (4) examine the effects of geographic, socio-economic, age and sex variation.

## Methods

### Case definition

To optimize capture of people with PsA, two definitions were applied to the Clinical Practice Research Datalink (CPRD) GOLD dataset.

Participants were classified as having ‘definite PsA’ if they had received a diagnosis of PsA that had been recorded in their primary care records. Cases were identified based on previously published Read code lists, which have been shown to have high positive predictive value [85% (95% CI: 75.8–91.7%)] in UK Primary Care databases [8–13], and supplemented by code searches in the CPRD GOLD Browser.

Participants were classified as having ‘probable PsA’ if they did not have a PsA diagnosis in their primary care records during the study window but were, based on their diagnosis and treatment history as defined by expert clinical opinion (co-authors W.G.D., J.H. and K.L.H.), likely to have PsA. Two types of probable PsA were defined, to account for underdiagnosed and misdiagnosed PsA cases. For both, persons with PsO were identified using code lists developed in previous studies of PsO [8, 9], and supplemented by code searches in the CPRD GOLD Browser to identify any additional codes referring to the same diagnosis. A list of drug substance names, created and reviewed by the clinical members of the study team, was translated into eligible product codes to identify individuals who had been prescribed treatment for PsA. Probable PsA was classified into type 1 and type 2 according to the following criteria.

Probable PsA type 1 refers to a diagnosis of PsO plus a diagnosis of arthritis [except seronegative RA (SN-RA), axial SpA (axSpA), and spinal arthritis] plus a record of at least one prescription for DMARD treatment used in PsA.

Probable PsA type 2 refers to a diagnosis of PsO plus a diagnosis of SN-RA, axSpA or spinal arthritis, irrespective of medication prescriptions.

No time restrictions, or additional exclusion criteria were applied to individuals who met these criteria. Alternative explanations for diagnoses were not explored. A full list of the identified Read codes is provided in Supplementary Data S1, available at *Rheumatology* online.

### Case identification

Definite and probable cases of PsA (hereafter ‘recorded cases of PsA’) were identified in the CPRD GOLD May 2021 release dataset [14]. CPRD GOLD is a large and broadly representative (based on age and sex) [15] database of anonymized UK primary care electronic medical records, which holds rich data on clinical diagnoses, symptoms and treatments and provides both individual- and practice-level data quality metrics. All available data for patients with at least one relevant Read code recorded from 1 January 1991 to 30 December 2020 (including data available prior to the start of our study window) was extracted.

Extracted data items were those needed to identify cases of PsA (see previous section, ‘Case definition’, and Supplementary Data S1, available at *Rheumatology* online). These included:

1. The diagnostic and product codes listed above.
2. Demographic data: year of birth [age at diagnosis was calculated from (year of diagnosis – year of birth) and categorized as young adults (18–29 years), younger working age (30–49 years), older working age (50–64 years), retirement age (65–79 years), older adults (80+ years)], sex (male/female), geographical region [based on general practitioner (GP) location], and region-specific Index of Multiple Deprivation (IMD, provided by CPRD as 1 = least deprived to 10 = most deprived, but re-coded for interpretability, with standard coding as 1 = most deprived to 10 = least deprived [16–19].
3. Quality control data: the date on which a practice was deemed to be of research quality or ‘Up To Standard’ and whether the participant was considered to have provided acceptable quality data after this date.

Definite PsA cases were assigned a diagnosis date of the first recorded PsA Read code during the study period, following the ‘up to standard’ date. Probable PsA cases were assigned a diagnosis date of the earliest date at which they satisfied the case definition.

Cases were excluded if they were <18 years on their date of diagnosis, were diagnosed after 31 December 31 2020, or were not considered to meet acceptable data quality standards.

### Modelling strategy

#### The observed annual prevalence and incidence of PsA

The prevalent cases within a given year were defined as the number of living adults aged ≥18 years diagnosed with PsA from the study start date to each calendar year of interest. The annual prevalence was calculated as the number of living PsA cases from 1 January 1991 to 31 December of the year of interest, divided by the adult mid-year population (i.e. the number of living persons aged ≥18 years on 1 July of the year of interest).

Incident PsA cases within a given year were defined as participants aged ≥18 years who were diagnosed with PsA for the first time ever, and who had registered with their GP at least 1 year prior to the date of diagnosis. Annual incidence rates were calculated as the number of new PsA cases between 1 January and 31 December, divided by the number of person-years at risk for each calendar year from 1991 to 2020. The person-years of follow-up were calculated for eligible people at risk (i.e. no previous diagnosis of PsA) from 1 January until the latest of transfer-out, last data collection, diagnosis of PsA, death, or 31 December of the study year.

#### The estimated annual prevalence and incidence of PsA in the UK

CPRD GOLD, while representative in terms of the age and sex of the UK population, has an estimated coverage of <10% [15], contains practices that are not evenly distributed in the UK, and which vary over time, and has an unknown representativeness for other factors of interest, such as IMD. For these reasons we standardized the observed estimates to the UK population using a Bayesian Multilevel Regression with a Post-stratification (MRP) approach. MRP is an effective method of adjusting the sample to be a more accurate representation of the population for a set of key variables [20–22].

The midyear UK adult population for each calendar year was obtained separately for the English regions (North East, North West, Yorkshire and The Humber, East Midlands, West Midlands, East, London, South East and South West), Northern Ireland, Scotland, and Wales, stratified by age group, sex and IMD decile. Data were obtained from a range of online sources and relevant government departments. A full list of sources is provided in Supplementary Data S2, available at *Rheumatology* online.

The standardization of the estimates was done in two stages. First, multilevel logistic regression and multilevel negative binomial regression models predicted, respectively, the prevalence and incidence of PsA, with varying intercepts for age, region, and IMD, and fixed effects for sex and years of follow-up. Second, the estimates for each stratification factor were weighted by the corresponding proportion of adults in the population. Full model specification and standardization details are shown in Supplementary Data S3, available at *Rheumatology* online. The analysis was undertaken using the rstan [23] and rstanarm [24] packages in R software version 4.2.2 [25].

### The impact of case misclassifications on annual prevalence estimates

Our use of multiple approaches for optimizing the capture of people with PsA, using unvalidated diagnosis codes, means that misclassification was possible. We tested for the impact of misclassification by extending the Bayesian multilevel regression model by specifying the prevalence as a function of the sensitivity and specificity of the case definition at either 87.5%, 77.8% or 63.6% of sensitivity and specificity. The full mathematical formulation and a description of prior elicitation is shown in Supplementary Data S3, available at *Rheumatology* online.

#### Patient and public involvement

This analysis was commissioned by *Vs* Arthritis. Patients and members of the public were not directly involved in this project, over and above any activities undertaken by *Vs* Arthritis prior to commissioning the work.

### Ethical approval

Informed written consent was not required for this study, as CPRD has Research Ethics Committee (REC) approval to enable CPRD to collect and share anonymized primary care data for observational research (REC reference: 05/MRE04/87). This study was approved by the Independent Scientific Advisory Committee (ISAC) responsible for applications to CPRD for data access (approval 20_000144).

## Results

### PsA cases in CPRD

A total of 26 716 patients satisfied the case definitions for PsA: 20 859 (78.1%) definite PsA, and 5857 (21.9%) probable PsA (4825 type 1; 1032 type 2). Two hundred and seventy-one patients (248 definite, 21 probable type 1 and 2 type 2) were <18 years (range 2–17 years) and were excluded. Of the remaining 26 445 cases, 93 (0.3%) had a date of diagnosis prior to 1 January 1991 and were included as prevalent cases only. A further 152 (0.6%) were diagnosed after 31 December 2020 and were excluded from the analysis. Of the remaining 26 293 PsA cases (21 137 incident cases during the follow-up window), 20 485 (77.9%) had definite PsA, and 5808 (22.1%) had probable PsA [4787 (18.2%) type 1; 1021 (3.9%) type 2]. Table 1 provides demographic data for all cases identified.

**Table 1.** Demographic data for all PsA cases identified in CPRD, and stratified by definition

			PsA definitions			
			All *N* = 26 293	Definite PsA *N* = 20 485	Probable PsA type 1^a^ *N* = 4787	Probable PsA type 2^b^ *N* = 1021
Sex	Male		12 522 (47.6)	10 167 (49.6)	1756 (36.7)	599 (58.7)
	Female		13 771 (52.4)	10 318 (50.4)	3031 (63.3)	422 (41.3)
Age at diagnosis	Young adults (18–29 years)		2506 (9.5)	2222 (10.8)	176 (3.7)	108 (10.6)
	Younger working age (30–49 years)		10 340 (39.3)	9150 (44.7)	799 (16.7)	391 (38.3)
	Older working age (50–64 years)		8418 (32.1)	6504 (31.8)	1623 (33.9)	291 (28.5)
	Retirement age (65–79 years)		4311 (16.4)	2364 (11.5)	1751 (36.6)	196 (19.2)
	Older adults (80+ years)		718 (2.7)	245 (1.2)	438 (9.1)	35 (3.4)
Geographical location	Northern Ireland		1806 (6.9)	1442 (7.0)	276 (5.8)	88 (8.6)
	Scotland		5847 (22.2)	4964 (24.3)	723 (15.1)	160 (15.7)
	Wales		3970 (15.1)	3029 (14.8)	777 (16.2)	164 (16.1)
	England		14 670 (55.8)	11 050 (53.9)	3011 (62.9)	609 (59.6)
		North East^c^	330 (2.2)	258 (2.3)	64 (2.1)	8 (1.3)
		North West^c^	2589 (17.6)	1822 (16.5)	655 (21.8)	112 (18.4)
		Yorkshire and The Humber^c^	616 (4.2)	415 (3.8)	174 (5.8)	27 (4.4)
		East Midlands^c^	592 (4.0)	437 (4.0)	131 (4.4)	24 (3.9)
		West Midlands^c^	1818 (12.4)	1333 (12.1)	413 (13.7)	72 (11.8)
		East of England^c^	1380 (9.4)	1023 (9.3)	293 (9.7)	64 (10.5)
		South West^c^	1724 (11.8)	1289 (11.7)	354 (11.8)	81 (13.3)
		London^c^	1485 (10.1)	1154 (10.4)	272 (9.0)	59 (9.7)
		South East Coast^c^	4136 (28.2)	3319 (30.0)	655 (21.8)	162 (26.6)

All values are *n* (%).

a Psoriasis (PsO) plus arthritis diagnosis plus DMARD treatment.

b PsO plus arthritis diagnosis (seronegative RA, axial SpA, or axial arthritis).

c Proportion of all persons identified with all types of PsA during the study period based in England (*n* = 14 670).

### The prevalence of PsA

**Figure 1.**
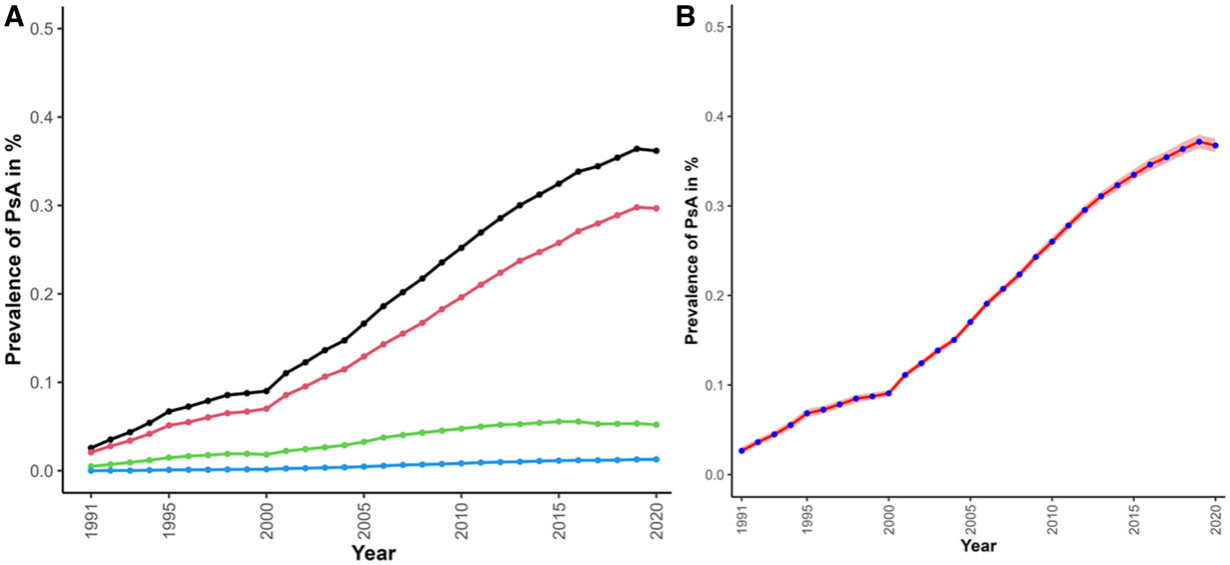
Observed and standardized (estimated) prevalence of PsA in the UK 1991–2020. (**A)** Observed prevalence of PsA by case definition type {black: all cases, red: definite PsA, green: probable PsA type 1 [psoriasis (PsO) plus arthritis diagnosis plus DMARD treatment], blue: probable PsA type 2 [PsO plus arthritis diagnosis (seronegative RA, axial SpA, or axial arthritis)]}. (**B**) Standardized (estimated) prevalence of PsA (all cases) over time [blue circles with line of best fit and 95% CI in red, against observed cases (black diamonds)]

In 1991, 195 prevalent cases of recorded PsA were observed among 757 132 individuals, an observed prevalence of 0.03% (Fig. 1, panel A and Supplementary Table S1, available at *Rheumatology* online). This had increased to 10 287 prevalent cases of recorded PsA among 2 843 402 individuals in 2020, an observed prevalence of 0.36% (Fig. 1 and Supplementary Table S2, available at *Rheumatology* online). This equated to a 12-fold increase in prevalence of recorded PsA over the study period. The estimated (standardized) prevalence of recorded PsA showed the same 12-fold increase, increasing from 0.03 in 1991 to 0.37 in 2020 (Fig. 1; Supplementary Table S3, available at *Rheumatology* online). The standardized prevalence stratified by age, sex, IMD, and geographical region are presented in Supplementary Tables S4–S7, available at *Rheumatology* online.

Within years and over time, standardized rates were consistently similar for men and women [prevalence (%) in 1991 cf. 2020: 0.03 cf. 0.36 for men and 0.03 cf. 0.38 for women]. There was an inverted U-shaped association with age, being lowest in the youngest (18–29 years) and oldest (80+ years) age groups and peaking in those aged 50–64 years (Supplementary Tables, available at *Rheumatology* online). There were no clear patterns of prevalence across IMD deciles, with similar magnitudes of increase over time across all deciles. When stratified by region, the 1991 prevalence was lowest in London (0.02%) and highest in Northern Ireland (0.04%). These differences were maintained over time (Supplementary Tables and Supplementary Figs S1–S4, available at *Rheumatology* online).

### Sensitivity analysis

Assuming no misclassification in identified cases, the estimated (standardized) prevalence of PsA in 2020 was 0.37% (0.36–0.37, deemed a credible interval). A sensitivity and specificity of the study case definitions of 87.5% resulted in a prevalence estimate of 0.45% (0.33–0.70). A sensitivity and specificity of the study case definitions of 77.8% resulted in a prevalence estimate of 0.48% (0.34–0.81). A sensitivity and specificity of the study case definitions of 63.6% resulted in a prevalence estimate of 0.58% (0.37–1.02).

### The incidence of PsA

**Figure 2.**
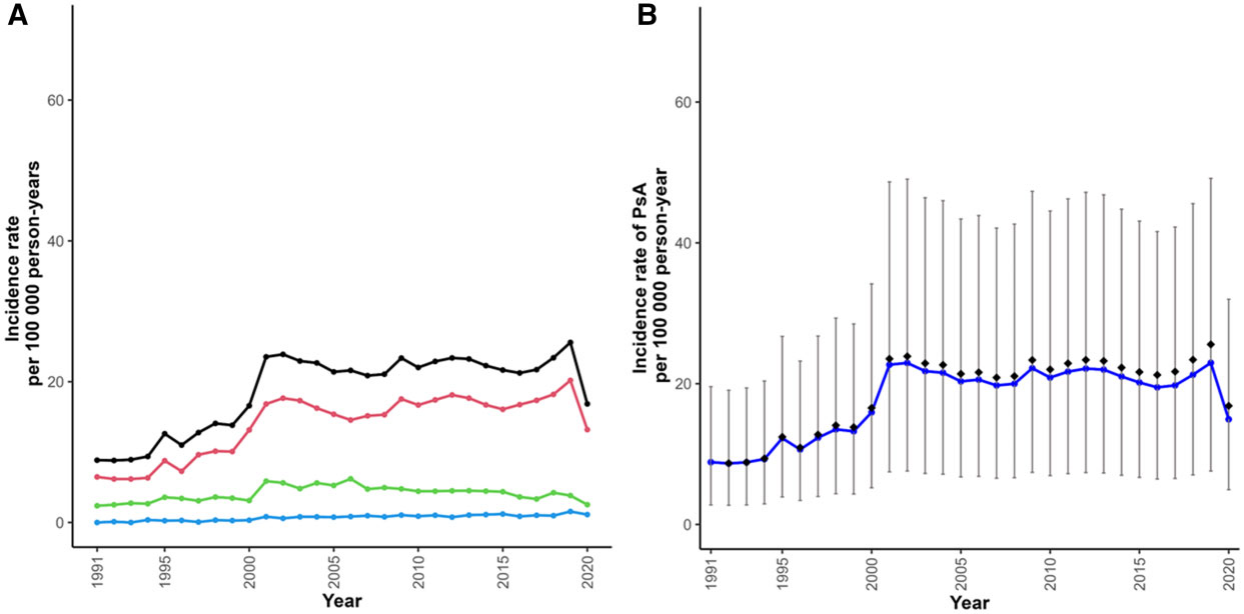
Observed and standardized (estimated) incidence of PsA in the UK 1991–2020. (**A**) Observed incidence of PsA, by case definition type {black: all cases, red: Definition 1 (definite PsA), green: Definition 2 [probable PsA: psoriasis (PsO) plus arthritis diagnosis plus DMARD treatment], blue: Definition 3: probable PsA [PsO plus arthritis diagnosis (seronegative RA, axial SpA, or axial arthritis)]. (**B**) estimated (standardized) incidence of PsA (all cases) over time (blue line with 95% CI), against observed cases (red diamonds)

In 1991, 67 recorded incident cases of PsA were observed (49 definite, 18 probable type 1, and 3 probable type 2) among 757 132 person-years of follow-up, an incidence of 8.85/100 000 person-years (Fig. 2, panel A; Supplementary Table S8, available at *Rheumatology* online). In 2020, 479 recorded incident cases were observed (375 definite, 72 probable type 1, and 32 probable type 2) among 2 843 402 person-years of follow-up, an observed recorded incidence of 16.85/100 000 person-years (Fig. 2, panel A; Supplementary Table S9, available at *Rheumatology* online). This equated to an almost 2-fold increase. The standardized incidence increased from 8.95/100 000 person years (95% CI 2.80–19.80) in 1991 to 15.08/100 000 person-years (5.02–32.32) in 2020 (Fig. 2, panel B), an almost 70% increase. The estimated (standardized) incidence stratified by age, sex, IMD, and geographical region are presented in Supplementary Tables S10–S14, available at *Rheumatology* online.

The standardized incidence of recorded PsA was similar for men and women, with a 70% increase from 1991 [men: 8.44 (2.66, 18.53); women: 9.47 (2.98, 20.80)] through to 2020 [men: 14.06 (4.75, 29.77); women: 15.76 (5.32, 33.39)] (Supplementary Table S9, available at *Rheumatology* online). Across all years of the study, the recorded PsA incidence was lowest in the youngest (18–29 years) and oldest (80+ years) age groups and peaked in those aged 50–64 years. There was no consistent relationship between IMD and incidence of recorded PsA over time. Within and across years of follow-up, the incidence of PsA was consistently lowest in London and highest in Northern Ireland (Supplementary Tables and Supplementary Figs S5–S8, available at *Rheumatology* online).

## Discussion

Between 1991 and 2020, the annual prevalence of recorded PsA in the UK has increased, while the annual incidence has remained constant after an initial increase between 1991 and 2001. Estimates were comparable between men and women, varied by age and geography, and had no consistent patterns of association with IMD. Here, ∼80% of recorded PsA cases were captured by PsA-specific Read codes. A minority were patients satisfying algorithms for ‘Probable PsA’. To our knowledge, no previous UK studies have included probable PsA, despite suggestions that PsA may be underdiagnosed and that diagnoses could be under-reported in primary care [6, 9]. We believe this work adds significant new knowledge to other papers in the area (e.g. Scott *et al*. [26], by extending the time-period under review, using a sensitive approach for identifying likely cases of PsA (i.e. probable PsA cases), and expanding the geographical regions within our estimates to include national and regional estimates.

There are limitations that should be noted. First, it is likely that we have not identified all cases of PsA. Misclassification of PsA due to variation in GP coding between practices is likely [9]. Further misclassification may result from medication data being based on prescription rather than dispensing data. Medication data may be missing if their presence in the primary care record is dependent on receipt and coding of information from secondary care (e.g. biologics). In the absence of linked primary and secondary care data, we could not ascertain how complete the primary care records were, or replace missing data with information from secondary care. Such linkage, while potentially beneficial, is not readily available and was not within the scope of this study. Data completeness may also have been impacted by temporal changes at practice level and nationally (e.g. changing availability of screening tools, or the establishment of new diagnostic criteria [6]). Second, cases of probable PsA were not validated and may have been misclassified (e.g. identifying cases on the basis of a prescription for a DMARD used in PsA may have incorrectly classified those who were using DMARDs to manage PsO, leading to over-estimation). However, we note that the probable PsA cases do not appear to drive the patterns of increasing prevalence and incidence. Further, the sensitivity analysis designed to investigate this demonstrated that, even at the highest level of misclassification, the rates in this study sat within those of previous estimates (0.3–1%) [2].

Third, although our Bayesian approach accounted for systematic differences in age, sex and IMD between CPRD and the UK population, systematic differences in ethnicity, a potentially important variable, were not explored. Ethnicity data in CPRD is available for an estimated 27% of all patients between 1990 and 2012 [27]. Here, we were able to classify the ethnicity of 49.7% of PsA cases [96.1% were white, which was higher than in the UK Census (in which the figure ranged from 93% in 1991 to 82% in 2021 [28, 29]). Regardless, ethnicity is not included in the denominator files provided by CPRD and could not be used in the study modelling strategy.

Finally, the date of probable PsA ‘diagnosis’ was defined as the latest date on which all criteria were satisfied. This could have inflated the rates of cases in the later years of the study window as the diagnoses accumulated. However, this approach was based on evidence that ∼90% of patients present with PsO or arthritis before a PsA diagnosis is made [9], and reflects clinician behaviour (the accumulation of diagnoses over time informs the diagnosis of PsA). The average interval between the first recorded relevant Read code and the latest relevant Read code was 5.9 years (probable type 1: 6.0 years, probable type 2: 5.4 years), with an increase from 3.8 years in 1991 to 6.9 years in 2020. Previous studies reported intervals between the first and second diagnoses of PsO and arthritis to be ∼8–10 years [9]. Our analysis strategy did not allow for exploration of the number of people who may have changed ‘diagnosis’ during or after the study window (i.e. who initially fulfilled probable PsA criteria and went on to receive a definite PsA diagnosis). We suggest this could be an interesting avenue for future research.

The prevalence estimates reported here are within the range of previous estimates (0.3–1%) and have a similar demographic distribution [2, 4–6, 8]. The results mirror evidence that the prevalence of psoriasis and PsA is increasing over time [30, 31]. We note our incidence estimates may appear to be higher than both global (8.26 per 100 000) and other European countries (6.0–8.0 per 100 000) [4, 32], which may reflect methodological differences in the way we conducted our studies. However, we also note that the CIs surrounding our estimates included those of previous studies, indicating that differences in estimates may reflect the expected uncertainty of estimating a relatively uncommon disease like PsA.

It has been hypothesized that the increased prevalence of these conditions is due to increased awareness, rather than a real increase [30, 31]. Indeed, given the extended time window under study here, these increases may represent improvements in identifying and diagnosing PsA, including the advent of specialist clinics, screening tools, and improved coding by GPs. Other causes may include changes to policy, and technology or care infrastructure (e.g. uptake of digital care records, and establishment of shared care practices), leading to changes in the way relevant diagnoses were recorded in primary care. However, others have argued that the observed increases in the prevalence and incidence of autoimmune diseases may be true increases, reflecting differences in risk factor exposure within populations, including socio-economic disparities, such as diet, smoking, air pollution, and other environmental factors [33].

For example, obesity, which is overrepresented in patients with PsA, may increase the risk of developing the disease, possibly related to a higher level of pro-inflammatory mediators [34], and is associated with increased disease activity [35]. Within the UK, variation and increases in obesity rates [36, 37] may also explain the differences in geographic patterns of PsA and the increases over time. Unfortunately, it was not possible to investigate the impact of obesity (or other potential risk factors) on these estimates due to issues of data availability. First, accurate and contemporary BMI data have not been available throughout the time window of our study; however, we note this has improved over time [38]. Second, similarly to ethnicity, BMI data were not included in the denominator files provided by CPRD and could therefore not be used in the study modelling strategy. Thus, the relative contributions of ‘true’ increases compared with ‘recorded’ increases cannot be delineated in our study.

In summary, the prevalence of recorded PsA in the UK has generally increased between 1991 and 2020, and the incidence within the same time window has remained stable following an increase from 1991 to 2001. The prevalence and incidence of recorded PsA were similar between men and women, and between age groups, and there were no clear patterns associated with deprivation.

## Supplementary Material

kead586_Supplementary_Data

## Data Availability

Clinical Practice Research Datalink (CPRD) data can be accessed with an appropriate licence from the CPRD and with approval from the Independent Scientific Advisory Committee. Licences are available from the CPRD, The Medicines and Healthcare Products Regulatory Agency, 10th Floor, 10 South Colonnade, Canary Wharf, London E14 4PU, England or http://www.cprd.com. The study documents, including the meta-data, analysis scripts, and output materials, are available on request from the corresponding author.
