# Supplementary material for: The epidemiology of psoriatic arthritis in the UK: a health intelligence analysis of UK Primary Care Electronic Health Records 1991–2020
Source: Rheumatology (Oxford). 2023 Nov 2;63(12):3346–52. doi: 10.1093/rheumatology/kead586 (PMC11636567; doi:10.1093/rheumatology/kead586)
Supplement: kead586_Supplementary_Data [file kead586_supplementary_data.zip › kead586_Supplementary_Data/rhe-23-1006-File004.docx]

**Supplementary Data S1 – Readcodes, medcodes and prodcodes used to identify individuals of interest.**

Codes to identify individuals with PsA, PsO and Arthritis, or PsO and Sero-negative (Rheumatoid) arthritis and axial spondyloarthritis.

The codes listed here were used both for extraction and classification of PsA. The data of any individuals with at least one of the below Readcodes was extracted from CPRD GOLD. Application of the case definitions then identified those who met our criteria for caseness*.

*individuals with PsO and Arthritis were also required to be in receipt of a PsA related medication

| Supplementary Table S1 – Readcodes and medcodes applied to the CPRD GOLD Clinical Table | | |
| --- | --- | --- |
| Term | GOLD Readcode | GOLD Medcode |
| PsA |  |  |
| Psoriatic arthropathy | M160.00 | 476 |
| Psoriatic arthritis | M160.11 | 96880 |
| Psoriatic arthropathy NOS | M160z00 | 12500 |
| Distal interphalangeal psoriatic arthropathy | M160100 | 32149 |
| Psoriasis spondylitica | M160000 | 26368 |
| [X]Other psoriatic arthropathies | Nyu1300 | 59107 |
| Arthritis mutilans | M160200 | 21503 |
| PsO | | |
| [X]Other psoriasis | Myu3000 | 66711 |
| Chronic large plaque psoriasis | M161F11 | 93511 |
| Erythrodermic psoriasis | M161H00 | 17094 |
| Flexural psoriasis | M161J00 | 107494 |
| Guttate psoriasis | M161600 | 3193 |
| H/O: psoriasis | 14F2.00 | 3437 |
| Other psoriasis | M161.00 | 22501 |
| Other psoriasis and similar disorders | M16y.00 | 41149 |
| Palmoplantar pustular psoriasis | M166.00 | 105229 |
| Psoriasis and similar disorders | M16..00 | 3733 |
| Psoriasis and similar disorders NOS | M16z.00 | 30975 |
| Psoriasis annularis | M161100 | 21104 |
| Psoriasis circinata | M161200 | 30272 |
| Psoriasis diffusa | M161300 | 42008 |
| Psoriasis discoidea | M161400 | 18755 |
| Psoriasis geographica | M161500 | 21633 |
| Psoriasis gyrate | M161700 | 65839 |
| Psoriasis inveterata | M161800 | 48257 |
| Psoriasis NOS | M161z00 | 172 |
| Psoriasis ostracea | M161900 | 60169 |
| Psoriasis palmaris | M161A00 | 8014 |
| Psoriasis plantaris | M161B00 | 2945 |
| Psoriasis punctata | M161C00 | 24136 |
| Psoriasis universalis | M161E00 | 20222 |
| Psoriasis unspecified | M161000 | 162 |
| Psoriasis vulgaris | M161F00 | 30210 |
| Pustular psoriasis | M161D00 | 4231 |
| Scalp psoriasis | M16y000 | 11761 |
| Arthritis | | |
| Acute arthritis | N06zA00 | 11269 |
| Arthritis | N06z.11 | 587 |
| Arthritis associated with other disease, 1st MTP joint | N03xH00 | 72011 |
| Arthritis associated with other disease, acromioclavic joint | N03x200 | 92962 |
| Arthritis associated with other disease, ankle | N03xD00 | 96187 |
| Arthritis associated with other disease, hip | N03x900 | 73637 |
| Arthritis associated with other disease, IP joint of toe | N03xK00 | 105650 |
| Arthritis associated with other disease, knee | N03xB00 | 62037 |
| Arthritis associated with other disease, lesser MTP joint | N03xJ00 | 111527 |
| Arthritis associated with other disease, MCP joint | N03x600 | 57901 |
| Arthritis associated with other disease, other tarsal joint | N03xG00 | 99026 |
| Arthritis associated with other disease, iliac joint | N03xA00 | 100356 |
| Arthritis associated with other disease, sternoclavic joint | N03x100 | 94590 |
| Arthritis associated with other disease, subtalar joint | N03xE00 | 96400 |
| Arthritis associated with other disease, talonavicular joint | N03xF00 | 73751 |
| Arthritis associated with other disease, wrist | N03x500 | 73928 |
| Arthritis associated with other disease, shoulder | N03x000 | 99997 |
| Arthritis associated with other disease, dist rad-uln joint | N03x400 | 73413 |
| Arthritis associated with other disease, PIP joint of finger | N03x700 | 73716 |
| Arthritis associated with other disease, DIP joint of finger | N03x800 | 73774 |
| Arthritis of spine | N11..11 | 2294 |
| Arthropathies NOS | N0z..00 | 3677 |
| Arthropathy NOS | N06z.00 | 2183 |
| Arthropathy NOS | N06zz00 | 25736 |
| Arthropathy NOS, of multiple sites | N06z900 | 23934 |
| Arthropathy NOS, of other specified site | N06z800 | 11742 |
| Arthropathy NOS, of the ankle and foot | N06z700 | 26382 |
| Arthropathy NOS, of the hand | N06z400 | 23384 |
| Arthropathy NOS, of the lower leg | N06z600 | 24909 |
| Arthropathy NOS, of the pelvic region and thigh | N06z500 | 14733 |
| Arthropathy NOS, of the shoulder region | N06z100 | 33674 |
| Arthropathy NOS, of the upper arm | N06z200 | 43308 |
| Arthropathy NOS, of unspecified site | N06z000 | 53314 |
| Chronic arthritis | N06zB00 | 4652 |
| Elbow arthritis NOS | N06z211 | 16591 |
| Foot arthritis NOS | N06z712 | 3543 |
| Ankle arthritis NOS | N06z711 | 11257 |
| Hip arthritis NOS | N06z511 | 7334 |
| Hand arthritis NOS | N06z411 | 8969 |
| Shoulder arthritis NOS | N06z111 | 8990 |
| Knee arthritis NOS | N06z611 | 2852 |
| Generalised arthritis | N065A00 | 17230 |
| Inflammatory polyarthropathy | N04..11 | 20615 |
| Other specified arthropathies | N0y..00 | 33791 |
| Other specified arthropathy | N06yz00 | 63820 |
| Other specified arthropathy of multiple sites | N06y900 | 66723 |
| Other specified arthropathy of other specified site | N06y800 | 40314 |
| Other specified arthropathy of the ankle and foot | N06y700 | 65004 |
| Other specified arthropathy of the forearm | N06y300 | 56306 |
| Other specified arthropathy of the hand | N06y400 | 42408 |
| Other specified arthropathy of the lower leg | N06y600 | 70372 |
| Other specified arthropathy of the pelvic region and thigh | N06y500 | 44509 |
| Other specified arthropathy of the shoulder region | N06y100 | 28612 |
| Other specified arthropathy of the upper arm | N06y200 | 106913 |
| Other specified arthropathy of unspecified site | N06y000 | 42887 |
| Other and unspecified arthropathies | N06..00 | 37536 |
| Polyarthritis | N065z11 | 1670 |
| Unspecified polyarthropathy of multiple sites | N065900 | 35629 |
| Unspecified polyarthropathy of other specified site | N065800 | 53871 |
| Unspecified polyarthropathy of unspecified site | N065000 | 56322 |
| Unspecified polyarthropathy or polyarthritis | N065.00 | 25020 |
| Unspecified polyarthropathy or polyarthritis NOS | N065z00 | 35936 |
| Wrist arthritis NOS | N06z311 | 2474 |
| Sero-negative (Rheumatoid) arthritis and axial spondyloarthritis |  |  |
| Sero negative arthritis | N04y100 | 4578 |
| Sero negative polyarthritis | N04y111 | 10919 |
| Seronegative rheumatoid arthritis | N040P00 | 6916 |
| Axial spondyloarthritis | N11F.00 | 109023 |
| Ankylosing spondylitis | N100.00 | 2184 |
| Inflammatory spondylopathies | N10..00 | 34880 |
| Other inflammatory spondylopathies | N10y.00 | 44026 |
| Other inflammatory spondylopathies NOS | N10yz00 | 37892 |

Product codes used to identify individuals with PsO and arthritis receiving treatment for PsA.

*N.B – codes here not used for initial data extraction, but for further refinement based on algorithm definition of PsA. Codes listed here represent all codes available in browser for each Drug Substance Name.*

| Supplementary Table S2– Prodcodes applied to the CPRD GOLD Therapy Table, ordered chronologically by number of codes. | | | | | |
| --- | --- | --- | --- | --- | --- |
| Ixekizumab | Apremilast | Tofacitinib | Certolizumab pegol | Infliximab | Secukinumab |
| 79879 | 64626 | 70198 | 43703 | 22392 | 73582 |
| 75975 | 78717 | 75417 | 68740 | 64636 | 68904 |
|  | 64612 | 74903 | 69393 | 16822 | 71450 |
|  |  |  | 44100 | 67558 | 67917 |
|  |  |  |  |  | 70351 |
|  |  |  |  |  |  |
| Abatacept | Golimumab | Rituximab | Tocilizumab | Ustekinumab | Leflunomide |
| 60030 | 47740 | 74553 | 62873 | 73378 | 18460 |
| 68419 | 62618 | 36294 | 56962 | 58174 | 71099 |
| 65586 | 68360 | 39111 | 67985 | 46309 | 16522 |
| 67916 | 47398 | 73503 | 46348 | 68359 | 4971 |
| 78069 | 46370 | 72094 | 62957 | 70171 | 68672 |
| 58194 | 60284 | 28490 | 75617 | 68383 | 17642 |
|  |  | 47502 | 41502 | 67585 | 73648 |
|  |  |  |  | 54485 | 67739 |
|  |  |  |  | 67983 | 48217 |
|  |  |  |  |  | 68671 |
|  |  |  |  |  | 79228 |
|  |  |  |  |  | 62007 |
|  |  |  |  |  | 4970 |
|  |  |  |  |  | 62993 |
|  |  |  |  |  | 72645 |
|  |  |  |  |  | 74213 |
|  |  |  |  |  | 6934 |
|  |  |  |  |  |  |
|  |  |  |  |  |  |
|  |  |  |  |  |  |
|  |  |  |  |  |  |
| Adalimumab | Etanercept | Sulfasalazine | Azathioprine | Methotrexate | Prednisolone |
| 67976 | 15921 | 34894 | 14395 | 71967 | 78144 |
| 76816 | 70538 | 31949 | 58654 | 76369 | 10934 |
| 50121 | 41058 | 33682 | 79083 | 32111 | 64221 |
| 52833 | 77888 | 53989 | 53797 | 72495 | 25272 |
| 50996 | 26387 | 4418 | 61160 | 70119 | 41515 |
| 71626 | 49856 | 72944 | 29340 | 74593 | 58234 |
| 56593 | 36556 | 3697 | 72980 | 61419 | 60421 |
| 75300 | 47843 | 10211 | 19072 | 61137 | 64007 |
| 6882 | 50998 | 7497 | 451 | 69850 | 32803 |
| 68836 | 72477 | 15373 | 34687 | 17035 | 34393 |
| 79923 | 14886 | 20862 | 671 | 71283 | 58987 |
| 72795 | 35126 | 359 | 41620 | 61178 | 79930 |
| 56972 | 66774 | 75546 | 36792 | 71898 | 2704 |
| 76397 | 56580 | 56909 | 571 | 21753 | 64008 |
| 23850 | 36008 | 2920 | 55858 | 57441 | 34631 |
| 77842 | 19257 | 42178 | 68643 | 61171 | 64128 |
| 76674 | 77594 | 79027 | 32101 | 61081 | 53336 |
| 48660 | 61373 | 58671 | 51181 | 30780 | 78546 |
| 69527 | 35419 | 31683 | 270 | 73401 | 61052 |
| 75882 | 69500 | 49244 | 13320 | 56037 | 59229 |
| 76716 | 50494 | 370 | 30495 | 59538 | 34748 |
|  |  | 55395 | 35518 | 77558 | 73294 |
|  |  | 60606 | 67421 | 58885 | 34914 |
|  |  | 71054 | 31215 | 60979 | 34978 |
|  |  | 1566 | 59006 | 58303 | 2368 |
|  |  | 31667 | 54982 | 72452 | 34221 |
|  |  | 49243 | 42988 | 61169 | 66645 |
|  |  | 61084 | 79370 | 49951 | 578 |
|  |  | 33968 | 34451 | 61082 | 28376 |
|  |  | 59099 | 75698 | 70674 | 65020 |
|  |  | 380 | 39115 | 72496 | 34781 |
|  |  | 13321 | 75063 | 36849 | 76020 |
|  |  | 4978 | 770 | 41585 | 80110 |
|  |  | 17880 | 43077 | 61122 | 66015 |
|  |  | 5427 | 22982 | 79193 | 64009 |
|  |  | 23401 | 1899 | 51120 | 34109 |
|  |  | 71048 | 43562 | 61796 | 34452 |
|  |  | 44183 | 12339 | 61151 | 44 |
|  |  | 34473 | 79321 | 57174 | 5913 |
|  |  | 11767 | 75012 | 36800 | 45302 |
|  |  | 54209 | 65339 | 62421 | 72421 |
|  |  | 14054 | 77940 | 78297 | 31532 |
|  |  | 62596 | 66003 | 61140 | 38407 |
|  |  | 71046 | 21899 | 61050 | 67076 |
|  |  | 75002 | 77123 | 61180 | 78129 |
|  |  | 508 | 73814 | 823 | 28859 |
|  |  | 78461 | 78478 | 72300 | 28375 |
|  |  | 77393 | 55773 | 72453 | 63791 |
|  |  | 61726 | 52921 | 72045 | 61162 |
|  |  |  | 34816 | 79194 | 67107 |
|  |  |  | 68977 | 50950 | 75763 |
|  |  |  | 26261 | 75803 | 73553 |
|  |  |  | 41670 | 44908 | 59338 |
|  |  |  | 74812 | 70059 | 21417 |
|  |  |  | 53869 | 69639 | 54434 |
|  |  |  | 63121 | 18424 | 65626 |
|  |  |  | 53956 | 70977 | 34404 |
|  |  |  |  | 70046 | 9727 |
|  |  |  |  | 34929 | 69568 |
|  |  |  |  | 61273 | 67507 |
|  |  |  |  | 73677 | 55480 |
|  |  |  |  | 41104 | 5490 |
|  |  |  |  | 28041 | 51753 |
|  |  |  |  | 76367 | 66550 |
|  |  |  |  | 61488 | 54118 |
|  |  |  |  | 72486 | 63082 |
|  |  |  |  | 61211 | 69811 |
|  |  |  |  | 68955 | 95 |
|  |  |  |  | 53385 | 56891 |
|  |  |  |  | 71453 | 61132 |
|  |  |  |  | 79422 | 63066 |
|  |  |  |  | 52606 | 32835 |
|  |  |  |  | 877 | 67559 |
|  |  |  |  | 62833 | 34461 |
|  |  |  |  | 61172 | 59912 |
|  |  |  |  | 35752 | 74239 |
|  |  |  |  | 61181 | 557 |
|  |  |  |  | 75868 | 66914 |
|  |  |  |  | 20951 | 79091 |
|  |  |  |  | 13428 | 33691 |
|  |  |  |  | 59723 | 58000 |
|  |  |  |  | 77901 | 55024 |
|  |  |  |  | 70411 | 23512 |
|  |  |  |  | 69928 | 69686 |
|  |  |  |  | 61085 | 820 |
|  |  |  |  | 59685 | 63549 |
|  |  |  |  | 71619 | 58369 |
|  |  |  |  |  | 63172 |
|  |  |  |  |  | 41745 |
|  |  |  |  |  | 29333 |
|  |  |  |  |  | 20095 |
|  |  |  |  |  | 80050 |
|  |  |  |  |  | 33990 |
|  |  |  |  |  | 53313 |
|  |  |  |  |  | 34660 |
|  |  |  |  |  | 75001 |
|  |  |  |  |  | 59283 |
|  |  |  |  |  | 33988 |
|  |  |  |  |  | 27962 |
|  |  |  |  |  | 64416 |
|  |  |  |  |  | 58384 |
|  |  |  |  |  | 73678 |
|  |  |  |  |  | 68497 |

**Supplementary Data S2 – General population data sources**

We obtained data on the number of persons aged >=18 years, separately for the English regions, Northern Ireland, Scotland, and Wales, stratified by age, sex and deprivation. We obtained data from the following sources:

**Scotland**

<https://www.gov.scot/publications/scottish-index-of-multiple-deprivation-2020v2-data-zone-look-up/>

<https://www.nrscotland.gov.uk/statistics-and-data/statistics/statistics-by-theme/population/population-estimates/2011-based-special-area-population-estimates/population-estimates-by-simd-2016>

**Northern Ireland**

<https://www.ninis2.nisra.gov.uk/public/SearchResults.aspx?sk=Usually;Resident;Population;single;year;age;sex;geographies>

<https://www.ninis2.nisra.gov.uk/public/Theme.aspx?themeNumber=10&themeName=People%20and%20Places>

<https://www.nisra.gov.uk/publications/nimdm17-soa-level-results>

**Wales**

<https://statswales.gov.wales/Catalogue/Community-Safety-and-Social-Inclusion/Welsh-Index-of-Multiple-Deprivation>

<https://www.ons.gov.uk/peoplepopulationandcommunity/populationandmigration/populationestimates/datasets/lowersuperoutputareamidyearpopulationestimates>

<https://www.ons.gov.uk/peoplepopulationandcommunity/populationandmigration/populationestimates/datasets/lowersuperoutputareamidyearpopulationestimates>

**England**

<https://www.ons.gov.uk/peoplepopulationandcommunity/populationandmigration/populationestimates/datasets/lowersuperoutputareamidyearpopulationestimates>

<https://www.ons.gov.uk/peoplepopulationandcommunity/populationandmigration/populationestimates/datasets/lowersuperoutputareamidyearpopulationestimates>

<https://www.gov.uk/government/statistics/english-indices-of-deprivation-2019>

<https://www.data.gov.uk/dataset/afc2ed54-f1c5-44f3-b8bb-6454eb0153d0/lower-layer-super-output-area-2001-to-lower-layer-super-output-area-2011-to-local-authority-district-2011-lookup-in-england-and-wales>

<https://digital.nhs.uk/data-and-information/publications/statistical/patients-registered-at-a-gp-practice/january-2021>

<https://geoportal.statistics.gov.uk/datasets/6a41affae7e345a7b2b86602408ea8a2_0/explore>

<https://www.england.nhs.uk/wp-content/uploads/2022/05/B1770-integrated-care-boards-establishment-order-2022.pdf>

**Supplementary Data S3 – Statistical analysis**

**Bayesian multilevel logistic regression**

**Prevalence model**

Let $Y_{j}$ and $n_{j}$ denotes the number of prevalent PsA cases and the total number of eligible individuals (denominator) in CPRD in the $j$^th^ stratification cells, respectively. The stratification cells are defined by the interaction of year, gender, age group, IMD, and regions. That is, the prevalent cases and denominator data were stratified on 2 sexes, 5 age categories, 10 IMD categories, 12 regions, and 30 years of follow-up, resulting in 36,000 stratification cells. The multilevel logistic regression model is then given by

$$Y_{j} \sim Binomial \left( n_{j},\theta_{j} \right),$$

$$\theta_{j} = logit^{-1}\left( \beta_{0}+\beta^{gender}*gender_{j}+\beta^{year}*year_{j}+\alpha_{age\left[ j \right]}^{age} +\alpha_{IMD\left[ j \right]}^{IMD}+\alpha_{region\left[ j \right]}^{region} \right),$$

with $\alpha_{age\left[ j \right]}^{age}\sim N\left( 0, \sigma^{age} \right), \alpha_{IMD\left[ j \right]}^{IMD}\sim N\left( 0, \sigma^{IMD} \right)$, and $\alpha_{region\left[ j \right]}^{region}\sim N\left( 0, \sigma^{region} \right)$, where $\theta_{j}$ is the prevalence of PSA in the j^th^ stratification cell. To complete, the Bayesian multilevel logistic regression model specification, we assumed non-informative priors for all model parameters. That is, we assign $normal^{+}(0, 0.5)$ priors for $\sigma^{age}, \sigma^{IMD},$ and $\sigma^{region}$ which allows the prevalence to vary moderately by these postratification factors. Further, a $Normal \left( 0, 2.5 \right)$ prior is assumed for all fixed effect terms ($\beta).$

**Incidence model**

Let $I_{j}$ and $P_{j}$ denotes the number of incident PsA cases and the total person-year at risk in the $j$^th^ stratification cells, respectively. The multilevel Negative-Binomial regression model is then given by

$$I_{j} \sim NB (P_{j}\lambda_{j} , \phi)$$

$\log\left( \lambda_{j} \right) =\log\left( P_{j} \right)+\beta_{0}+\beta^{gender}*gender_{j}+\beta^{year}*year_{j}+\alpha_{age\left[ j \right]}^{age} +\alpha_{IMD\left[ j \right]}^{IMD}+\alpha_{region\left[ j \right]}^{region}$,

where $\phi$ is the overdispersion parameter. As before, we assumed a non-informative prior specification to complete the Bayesian model.

**Post-stratification (standardization)**

Once the models are fitted using CPRD data and the prevalence and incidence rates are estimated for each stratification cells, we can obtain standardised prevalence and incidence rates (along with their 95% CI) for each stratification factor as follows

1. Standardized prevalence

$$\theta_{subgroup}^{MRP}= \frac{\sum_{j\in subgroup} N_{j}\theta_{j}}{\sum_{j\in subgroup} N_{j}},$$

where $N_{j}$ is the number of persons for the j^th^ stratification cell in the population.

1. Standardized incidence rate

$$\lambda_{subgroup}^{MRP}= \frac{\sum_{j\in subgroup} N_{j}\lambda_{j}}{\sum_{j\in subgroup} N_{j}},$$

where $N_{j}$ is the number of persons for the j^th^ stratification cell in the population.

**Bayesian multilevel logistic regression with misclassification**

**Prevalence model with misclassification**

As before, let $Y_{j}$ and $n_{j}$ denotes the number of prevalent PsA cases and the total number of eligible individuals (denominator) in CPRD in the $j$^th^ stratification cells, respectively. The multilevel logistic regression model with misclassification is then given by

$$Y_{j} \sim Binomial \left( n_{j},\theta_{j} \right),$$

$$\theta_{j}=\pi_{j}\delta+\left( 1-\pi_{j} \right)\left( 1-\gamma\right),$$

$$\pi_{j} = logit^{-1}\left( \beta_{0}+\beta^{gender}*gender_{j}+\beta^{year}*year_{j}+\alpha_{age\left[ j \right]}^{age} +\alpha_{IMD\left[ j \right]}^{IMD}+\alpha_{region\left[ j \right]}^{region} \right),$$

with $\alpha_{age\left[ j \right]}^{age}\sim N\left( 0, \sigma^{age} \right), \alpha_{IMD\left[ j \right]}^{IMD}\sim N\left( 0, \sigma^{IMD} \right)$, and $\alpha_{region\left[ j \right]}^{region}\sim N\left( 0, \sigma^{region} \right)$, where $\theta_{j}$ is the prevalence of PSA in the j^th^ CPRD stratification cell, $\pi_{j}$ is the true prevalence in the j^th^ stratification cell, $\delta$ and $\gamma$ are the sensitivity and specificity of the case definition, respectively. When $\delta$ and $\gamma$ are equals to 1 the above model is reduced to the standard multilevel regression model. Due to a lack of prior validation study, we were unable to specify the exact values of the sensitivity and specificity of the case definition. Instead, we specify a series of prior distributions reflecting varying levels of uncertainty on the case definitions. That is, we assume

$$\delta, \gamma\sim Beta\left( a, b \right).$$

We considered three prior specifications for sensitivity and specificity. We elicited the beta distribution parameters by finding $v_{1}$ and $v_{2}$ such that the absolute value of the difference between the cumulative distribution function (CDF) of the beta distribution evaluated at the upper boundary (ub) and lower boundary (lb) is as close as possible to the 95% confidence interval (ci). Mathematically, this is written as

$$\min_{v_{1}, v_{2}} |I_{ub}\left( v_{1}, v_{2} \right)-I_{lb}\left( v_{1},v_{2} \right)-ci|$$

Where $I_{ub}\left( v_{1}, v_{2} \right)$ and $I_{lb}\left( v_{1},v_{2} \right)$ are the CDF of a beta distribution. For three different values of $ub$ and $lb$, representing varying levels of misclassification, we obtained the following three beta prior specifications.

1. Beta (8, 5), representing 95% of sensitivity and specificity values lies between 0.35 ($lb$) and 0.85 ($ub$), with a most likely value of 0.636
2. Beta (8, 3), representing 95% of sensitivity and specificity values lies between 0.45 ($lb$) and 0.9 ($ub$)0, with a most likely value of 0.778 and
3. Beta (8, 2) representing 95% of sensitivity and specificity values lies between 0.50 ($lb$) and 0.95 ($ub$) with the most likely values of 0.875.

To guarantee the identifiability of the model, we assume sensitivity > 1 - specificity (i.e., a true positive rate higher than a false positive rate). Further, we considered a unit logistic prior to the centred $\beta_{0}+\beta^{gender}*\overline{gender}$corresponding to a uniform (0, 1) prior distribution for the average person in the UK to have PsA. For the remaining model parameters, we considered the same prior specifications as before.
