# Supplementary material for: The epidemiology of psoriatic arthritis in the UK: a health intelligence analysis of UK Primary Care Electronic Health Records 1991–2020
Source: Rheumatology (Oxford). 2023 Nov 2;63(12):3346–52. doi: 10.1093/rheumatology/kead586 (PMC11636567; doi:10.1093/rheumatology/kead586)
Supplement: kead586_Supplementary_Data [file kead586_supplementary_data.zip › kead586_Supplementary_Data/rhe-23-1006-File005.docx]

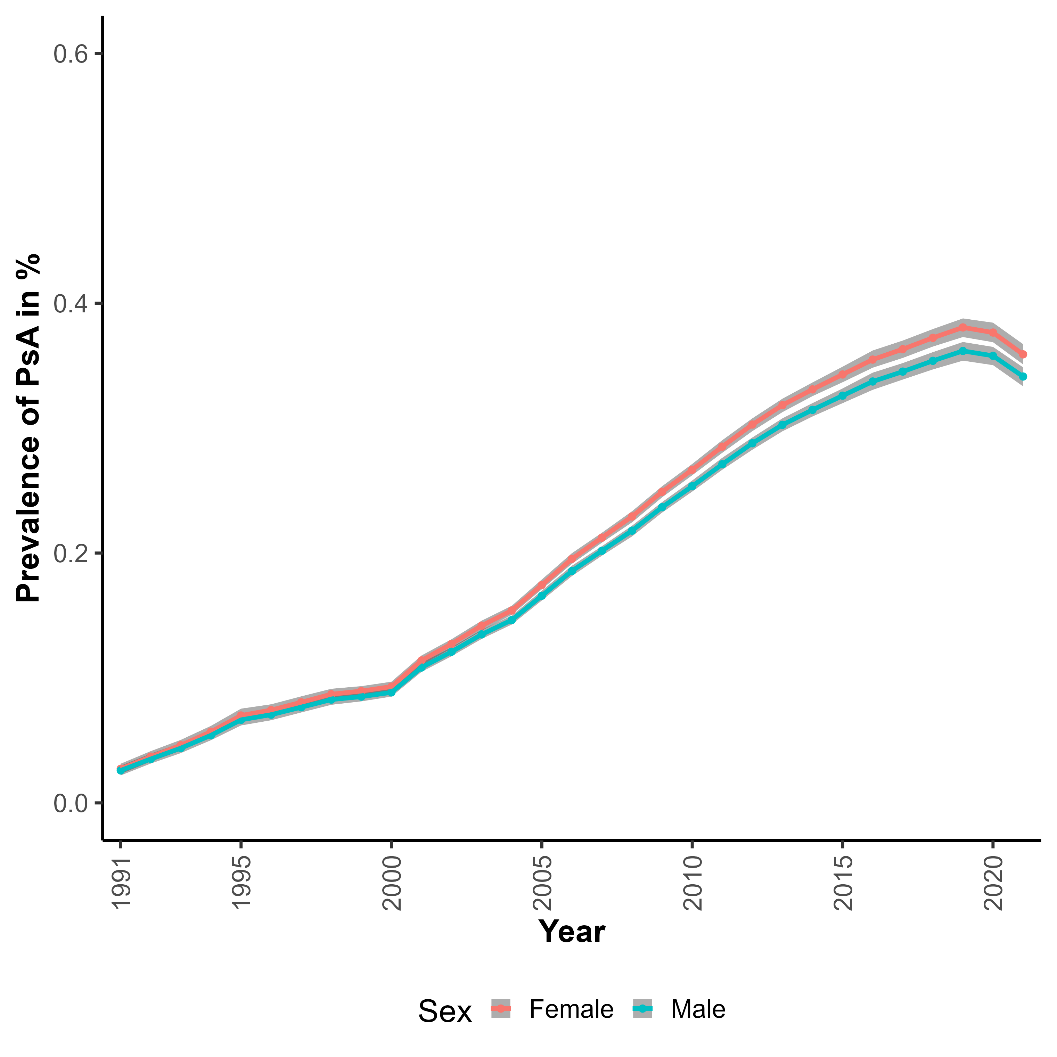


Supplementary Figure S1 - Estimated prevalence of PsA (all cases) over time by sex


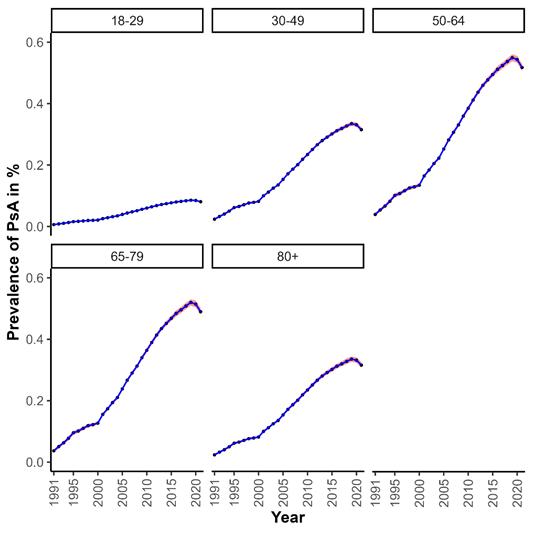


Supplementary Figure S2 - Estimated prevalence of PsA (all cases) over time by age


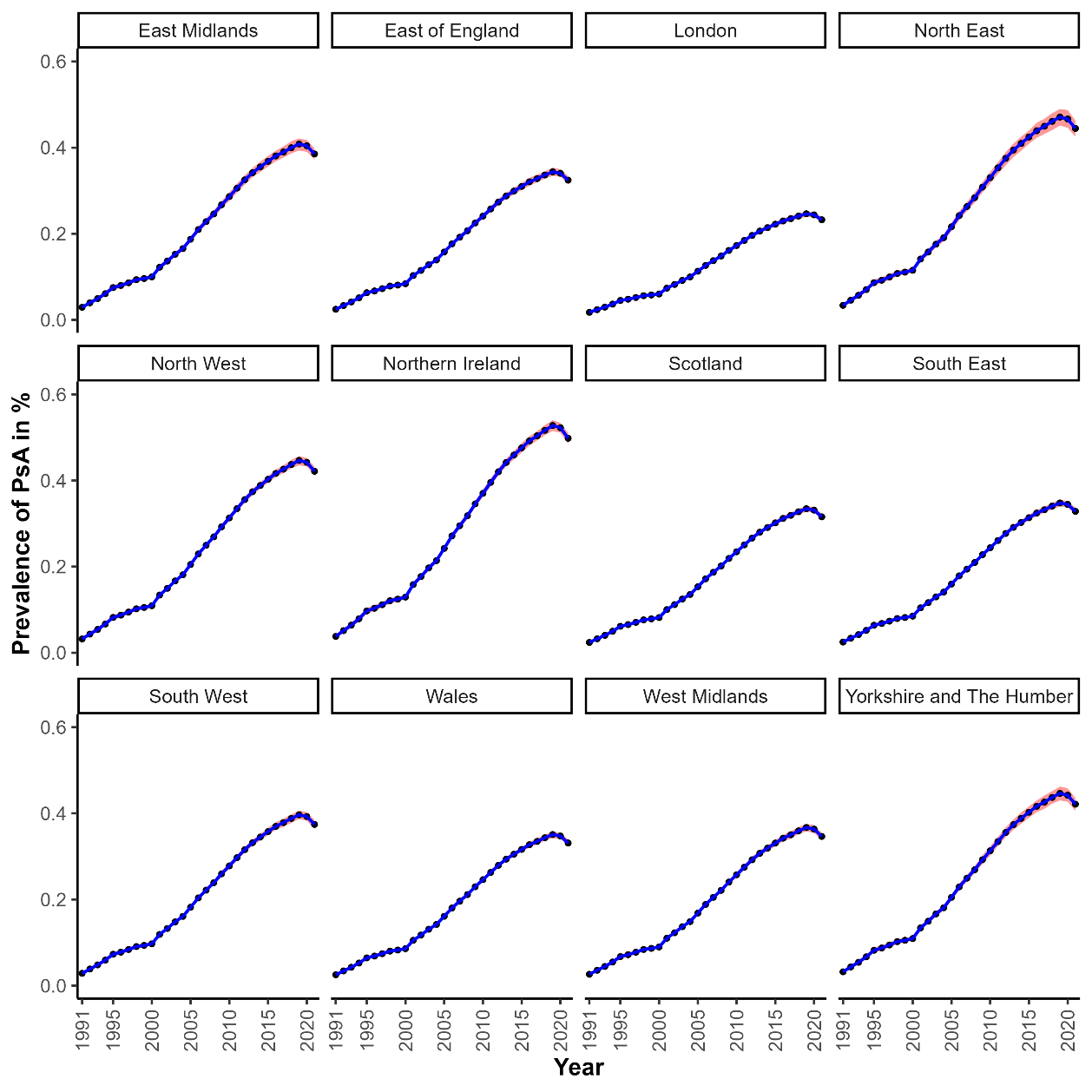

Supplementary Figure S3 - Estimated prevalence of PsA (all cases) over time by region


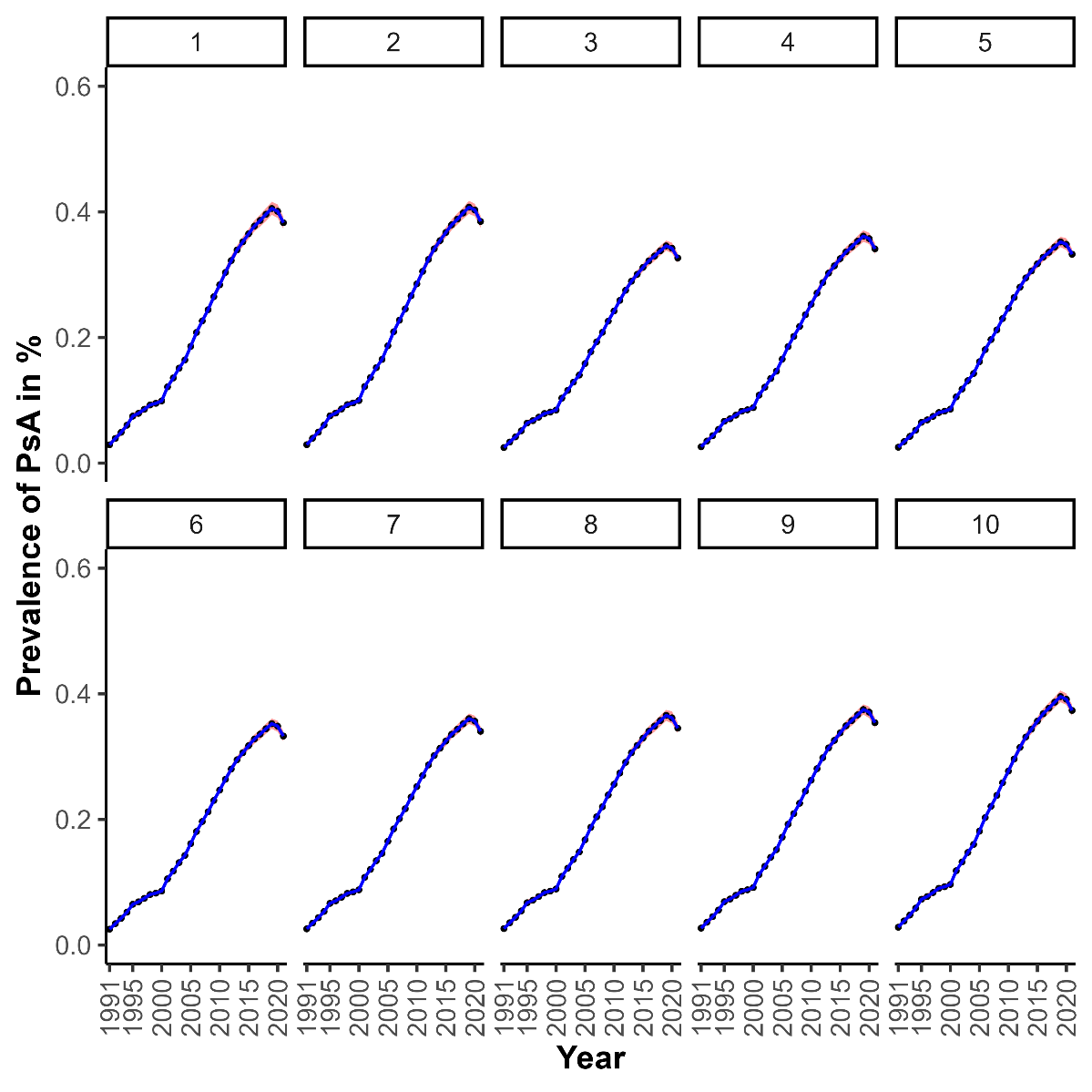


Supplementary Figure S4 - Estimated prevalence of PsA (all cases) over time by index of multiple deprivation decile (1=most deprived, 10=least deprived).


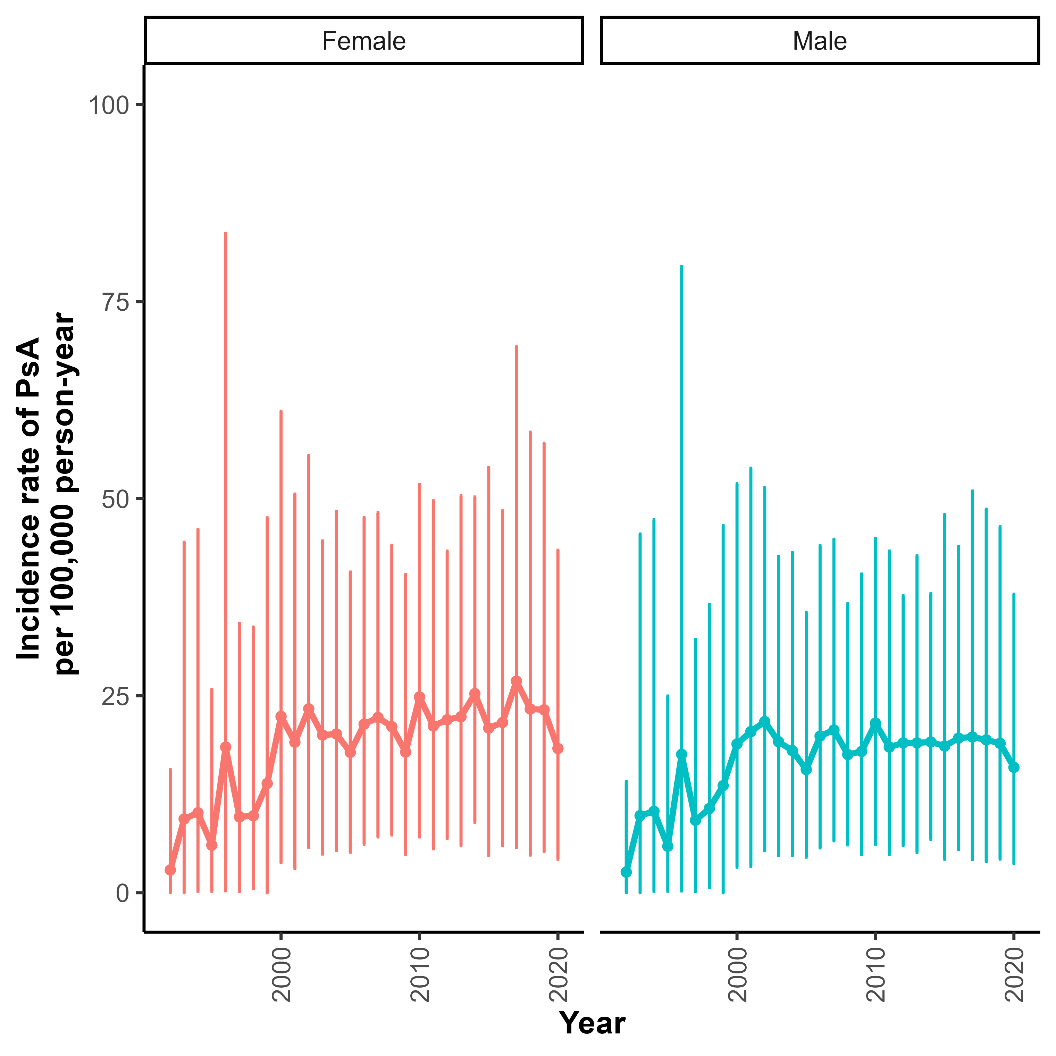


Supplementary Figure S5 - Estimated incidence of PsA (all cases) over time by sex


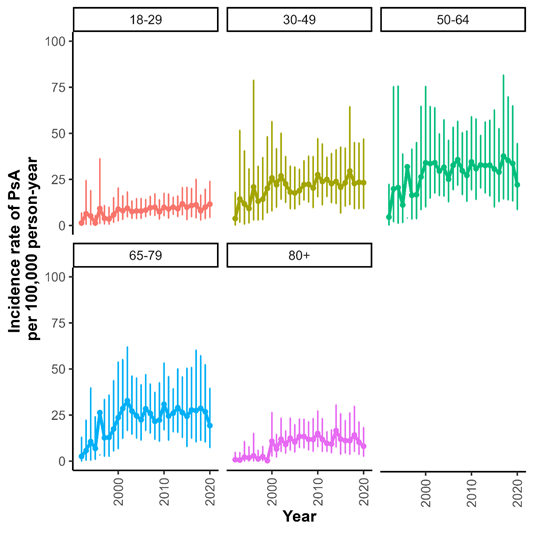


Supplementary Figure S6 - Estimated incidence of PsA (all cases) over time by age


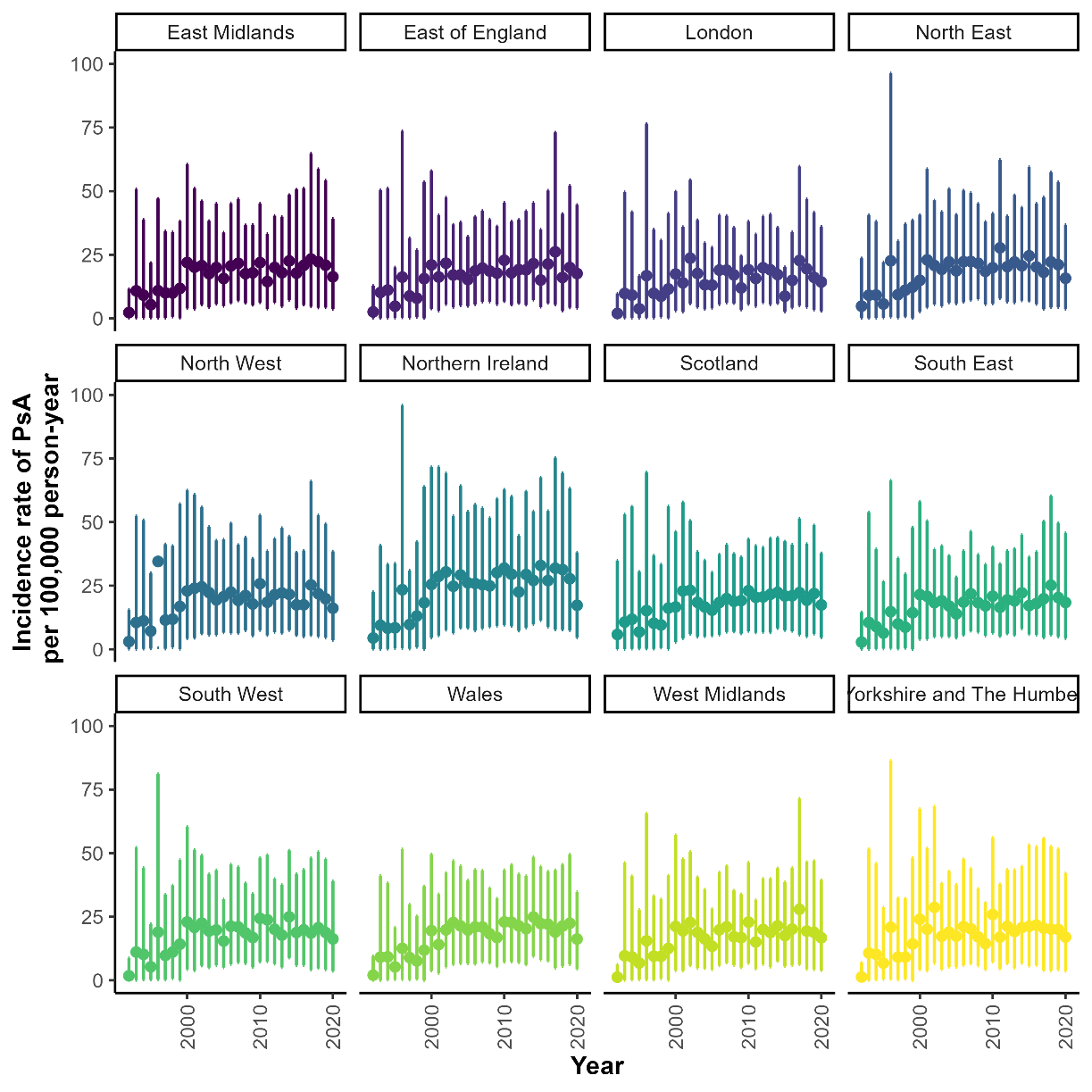

Supplementary Figure S7 - Estimated incidence of PsA (all cases) over time by region


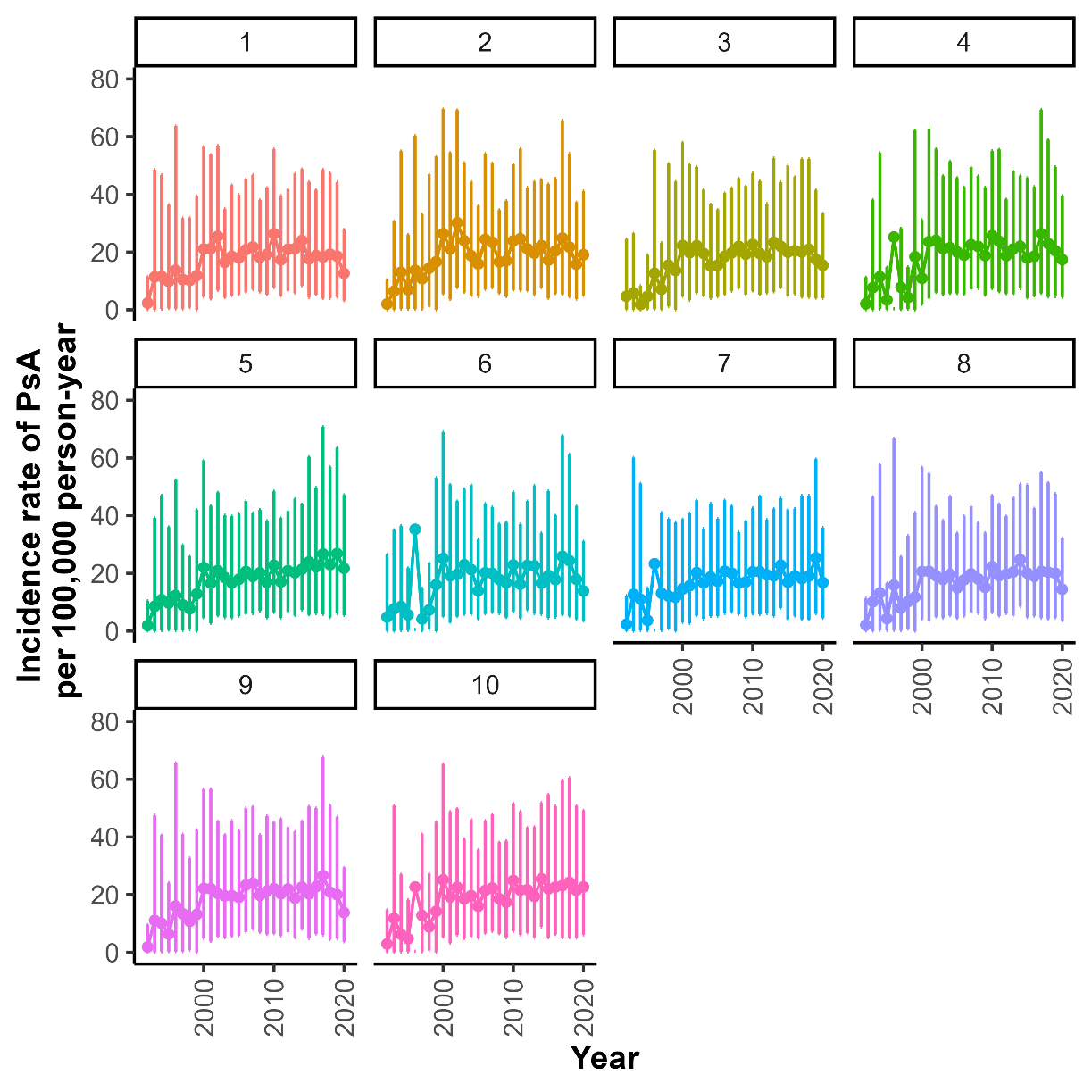


Supplementary Figure S8 - Estimated incidence of PsA (all cases) over time by index of multiple deprivation decile (1=most deprived, 10=least deprived).
