# Supplementary material for: The epidemiology of psoriatic arthritis in the UK: a health intelligence analysis of UK Primary Care Electronic Health Records 1991–2020
Source: Rheumatology (Oxford). 2023 Nov 2;63(12):3346–52. doi: 10.1093/rheumatology/kead586 (PMC11636567; doi:10.1093/rheumatology/kead586)
Supplement: kead586_Supplementary_Data [file kead586_supplementary_data.zip › kead586_Supplementary_Data/rhe-23-1006-File006.docx]

# Observed prevalence in CPRD in 1991 and 2020, all cases and stratified by case definition, sex, age, deprivation and region

| **Supplementary Table S1 - Observed prevalence of PsA in CPRD in 1991** | | | |
| --- | --- | --- | --- |
| Stratification variable | Cases | Denominator | Proportion |
| **Case definition** | | | |
| All | 195 | 757132 | 0.03 |
| Definition 1: Definite PsA | 158 | 757132 | 0.02 |
| Definition 2: Probable PsA* | 37 | 757132 | 0.00 |
| Definition 3: Probable PsA** |  |  |  |
| **Stratified by sex** | | | |
| Male | 102 | 392699 | 0.03 |
| Female | 93 | 364433 | 0.03 |
| **Stratified by age** | | | |
| 18-29 | 21 | 159187 | 0.01 |
| 30-49 | 84 | 276588 | 0.03 |
| 50-64 | 67 | 153956 | 0.04 |
| 65-79 | 22 | 125090 | 0.02 |
| 80+ | 1 | 42311 | 0.004 |
| **Stratified by IMD decile (1=most deprived, 10=least deprived).** | | | |
| 1 | 14 | 64801 | 0.02 |
| 2 | 14 | 34719 | 0.04 |
| 3 | 22 | 69798 | 0.03 |
| 4 | 9 | 47752 | 0.02 |
| 5 | 12 | 57941 | 0.02 |
| 6 | 11 | 88552 | 0.01 |
| 7 | 30 | 85738 | 0.03 |
| 8 | 29 | 95294 | 0.03 |
| 9 | 25 | 123727 | 0.02 |
| 10 | 29 | 88810 | 0.03 |
| **Stratified by region** | | | |
| East midlands | 13 | 68071 | 0.02 |
| East of England | 12 | 80978 | 0.01 |
| London | 10 | 57223 | 0.02 |
| North East | 12 | 18318 | 0.07 |
| North West | 30 | 102329 | 0.03 |
| Northern Ireland | 4 | 11127 | 0.04 |
| Scotland | 5 | 18020 | 0.03 |
| South East | 28 | 115216 | 0.02 |
| South West | 8 | 64785 | 0.01 |
| Wales | 28 | 79512 | 0.04 |
| West Midlands | 23 | 70303 | 0.03 |
| Yorkshire and The Humber | 22 | 71250 | 0.03 |

| **Supplementary Table S2 - Observed prevalence of PsA in CPRD in 2020** | | | |
| --- | --- | --- | --- |
| Stratification variable | Cases | Denominator | Proportion |
| **Case definition** | | | |
| All | 10287 | 2843402 | 0.36 |
| Definition 1: Definite PsA | 8437 | 2843402 | 0.30 |
| Definition 2: Probable PsA* | 1484 | 2843402 | 0.05 |
| Definition 3: Probable PsA** | 366 | 2843402 | 0.01 |
| **Stratified by sex** | | | |
| Male | 4773 | 1403285 | 0.34 |
| Female | 5514 | 1440117 | 0.38 |
| **Stratified by age** | | | |
| 18-29 | 257 | 486398 | 0.05 |
| 30-49 | 2716 | 956295 | 0.28 |
| 50-64 | 3953 | 725260 | 0.55 |
| 65-79 | 2695 | 492450 | 0.55 |
| 80+ | 666 | 182999 | 0.36 |
| **Stratified by IMD decile (1=most deprived, 10=least deprived).** | | | |
| 1 | 814 | 237995 | 0.34 |
| 2 | 1094 | 265737 | 0.41 |
| 3 | 953 | 277376 | 0.34 |
| 4 | 798 | 213016 | 0.37 |
| 5 | 982 | 264866 | 0.37 |
| 6 | 961 | 275401 | 0.35 |
| 7 | 1336 | 386726 | 0.35 |
| 8 | 897 | 259351 | 0.35 |
| 9 | 1317 | 379988 | 0.35 |
| 10 | 1135 | 282946 | 0.40 |
| **Stratified by region** | | | |
| East midlands*** |  | | |
| East of England | 25 | 12539 | 0.20 |
| London | 208 | 147738 | 0.14 |
| North East*** |  | | |
| North West | 465 | 164827 | 0.28 |
| Northern Ireland | 1129 | 237530 | 0.48 |
| Scotland | 3510 | 1155413 | 0.30 |
| South East | 631 | 211261 | 0.30 |
| South West | 98 | 37846 | 0.26 |
| Wales | 2083 | 769192 | 0.27 |
| West Midlands | 278 | 101812 | 0.27 |
| Yorkshire and The Humber | 10 | 5244 | 0.19 |

*Definition 2: Probable PsA: psoriasis (PsO) plus arthritis diagnosis plus disease modifying anti-rheumatic drug (DMARD) treatment **Definition 3: Probable PsA: PsO plus arthritis diagnosis (Seronegative-Rheumatoid Arthritis, Axial Spondyloarthritis or axial arthritis). ***observed data not available for these regions in 2020.

# Estimated prevalence between 1991 and 2020, all cases and stratified by case definition, sex, age, deprivation and region

| **Supplementary Table S3 – Annual estimates of PsA prevalence in the UK between 1991 and 2020** | | | | | | | | | | |  |  |  |  |  |  |  |
| --- | --- | --- | --- | --- | --- | --- | --- | --- | --- | --- | --- | --- | --- | --- | --- | --- | --- |
| Year | | Prevalence estimate | | | Lower confidence limit | | Upper confidence limit | | | |  |  |  |  |  |  |  |
| 1991 | | 0.03 | | | 0.02 | | 0.03 | | | |  |  |  |  |  |  |  |
| 1992 | | 0.04 | | | 0.03 | | 0.04 | | | |  |  |  |  |  |  |  |
| 1993 | | 0.04 | | | 0.04 | | 0.05 | | | |  |  |  |  |  |  |  |
| 1994 | | 0.06 | | | 0.05 | | 0.06 | | | |  |  |  |  |  |  |  |
| 1995 | | 0.07 | | | 0.06 | | 0.07 | | | |  |  |  |  |  |  |  |
| 1996 | | 0.07 | | | 0.07 | | 0.08 | | | |  |  |  |  |  |  |  |
| 1997 | | 0.08 | | | 0.07 | | 0.08 | | | |  |  |  |  |  |  |  |
| 1998 | | 0.09 | | | 0.08 | | 0.09 | | | |  |  |  |  |  |  |  |
| 1999 | | 0.09 | | | 0.08 | | 0.09 | | | |  |  |  |  |  |  |  |
| 2000 | | 0.09 | | | 0.09 | | 0.09 | | | |  |  |  |  |  |  |  |
| 2001 | | 0.11 | | | 0.11 | | 0.11 | | | |  |  |  |  |  |  |  |
| 2002 | | 0.12 | | | 0.12 | | 0.13 | | | |  |  |  |  |  |  |  |
| 2003 | | 0.14 | | | 0.14 | | 0.14 | | | |  |  |  |  |  |  |  |
| 2004 | | 0.15 | | | 0.15 | | 0.15 | | | |  |  |  |  |  |  |  |
| 2005 | | 0.17 | | | 0.17 | | 0.17 | | | |  |  |  |  |  |  |  |
| 2006 | | 0.19 | | | 0.19 | | 0.19 | | | |  |  |  |  |  |  |  |
| 2007 | | 0.21 | | | 0.20 | | 0.21 | | | |  |  |  |  |  |  |  |
| 2008 | | 0.22 | | | 0.22 | | 0.23 | | | |  |  |  |  |  |  |  |
| 2009 | | 0.24 | | | 0.24 | | 0.25 | | | |  |  |  |  |  |  |  |
| 2010 | | 0.26 | | | 0.26 | | 0.26 | | | |  |  |  |  |  |  |  |
| 2011 | | 0.28 | | | 0.27 | | 0.28 | | | |  |  |  |  |  |  |  |
| 2012 | | 0.30 | | | 0.29 | | 0.30 | | | |  |  |  |  |  |  |  |
| 2013 | | 0.31 | | | 0.31 | | 0.32 | | | |  |  |  |  |  |  |  |
| 2014 | | 0.32 | | | 0.32 | | 0.33 | | | |  |  |  |  |  |  |  |
| 2015 | | 0.33 | | | 0.33 | | 0.34 | | | |  |  |  |  |  |  |  |
| 2016 | | 0.35 | | | 0.34 | | 0.35 | | | |  |  |  |  |  |  |  |
| 2017 | | 0.35 | | | 0.35 | | 0.36 | | | |  |  |  |  |  |  |  |
| 2018 | | 0.36 | | | 0.36 | | 0.37 | | | |  |  |  |  |  |  |  |
| 2019 | | 0.37 | | | 0.36 | | 0.38 | | | |  |  |  |  |  |  |  |
| 2020 | | 0.37 | | | 0.36 | | 0.37 | | | |  |  |  |  |  |  |  |
|  | | | | | | | | | | |  |  |  |  |  |  |  |
| **Supplementary Table S4 – Annual estimates of PsA prevalence in the UK between 1991 and 2020 stratified by sex** | | | | | | | | | | | | | | | | |  |
| Year | | Women | | | | | | | | Men | | | | | | |  |
|  |  | Prevalence estimate | | | Lower confidence limit | | Upper confidence limit | | | Prevalence estimate | | | Lower confidence limit | | Upper confidence limit | |  |
| 1991 | | 0.03 | | | 0.02 | | 0.03 | | | 0.03 | | | 0.02 | | 0.03 | |  |
| 1992 | | 0.04 | | | 0.03 | | 0.04 | | | 0.04 | | | 0.03 | | 0.04 | |  |
| 1993 | | 0.05 | | | 0.04 | | 0.05 | | | 0.04 | | | 0.04 | | 0.05 | |  |
| 1994 | | 0.06 | | | 0.05 | | 0.06 | | | 0.05 | | | 0.05 | | 0.06 | |  |
| 1995 | | 0.07 | | | 0.07 | | 0.08 | | | 0.07 | | | 0.06 | | 0.07 | |  |
| 1996 | | 0.07 | | | 0.07 | | 0.08 | | | 0.07 | | | 0.07 | | 0.08 | |  |
| 1997 | | 0.08 | | | 0.08 | | 0.09 | | | 0.08 | | | 0.07 | | 0.08 | |  |
| 1998 | | 0.09 | | | 0.08 | | 0.09 | | | 0.08 | | | 0.08 | | 0.09 | |  |
| 1999 | | 0.09 | | | 0.09 | | 0.09 | | | 0.09 | | | 0.08 | | 0.09 | |  |
| 2000 | | 0.09 | | | 0.09 | | 0.10 | | | 0.09 | | | 0.09 | | 0.09 | |  |
| 2001 | | 0.11 | | | 0.11 | | 0.12 | | | 0.11 | | | 0.10 | | 0.11 | |  |
| 2002 | | 0.13 | | | 0.12 | | 0.13 | | | 0.12 | | | 0.12 | | 0.12 | |  |
| 2003 | | 0.14 | | | 0.14 | | 0.15 | | | 0.13 | | | 0.13 | | 0.14 | |  |
| 2004 | | 0.15 | | | 0.15 | | 0.16 | | | 0.15 | | | 0.14 | | 0.15 | |  |
| 2005 | | 0.17 | | | 0.17 | | 0.18 | | | 0.17 | | | 0.16 | | 0.17 | |  |
| 2006 | | 0.20 | | | 0.19 | | 0.20 | | | 0.19 | | | 0.18 | | 0.19 | |  |
| 2007 | | 0.21 | | | 0.21 | | 0.22 | | | 0.20 | | | 0.20 | | 0.21 | |  |
| 2008 | | 0.23 | | | 0.22 | | 0.23 | | | 0.22 | | | 0.21 | | 0.22 | |  |
| 2009 | | 0.25 | | | 0.24 | | 0.25 | | | 0.24 | | | 0.23 | | 0.24 | |  |
| 2010 | | 0.27 | | | 0.26 | | 0.27 | | | 0.25 | | | 0.25 | | 0.26 | |  |
| 2011 | | 0.29 | | | 0.28 | | 0.29 | | | 0.27 | | | 0.27 | | 0.28 | |  |
| 2012 | | 0.30 | | | 0.30 | | 0.31 | | | 0.29 | | | 0.28 | | 0.29 | |  |
| 2013 | | 0.32 | | | 0.31 | | 0.32 | | | 0.30 | | | 0.30 | | 0.31 | |  |
| 2014 | | 0.33 | | | 0.33 | | 0.34 | | | 0.31 | | | 0.31 | | 0.32 | |  |
| 2015 | | 0.34 | | | 0.34 | | 0.35 | | | 0.33 | | | 0.32 | | 0.33 | |  |
| 2016 | | 0.35 | | | 0.35 | | 0.36 | | | 0.34 | | | 0.33 | | 0.34 | |  |
| 2017 | | 0.36 | | | 0.36 | | 0.37 | | | 0.35 | | | 0.34 | | 0.35 | |  |
| 2018 | | 0.37 | | | 0.37 | | 0.38 | | | 0.35 | | | 0.35 | | 0.36 | |  |
| 2019 | | 0.38 | | | 0.37 | | 0.39 | | | 0.36 | | | 0.35 | | 0.37 | |  |
| 2020 | | 0.38 | | | 0.37 | | 0.38 | | | 0.36 | | | 0.35 | | 0.37 | |  |
|  | |  | | |  | |  | | |  | | |  | |  | |  |
| **Supplementary Table S5 – Annual estimates of PsA prevalence in the UK between 1991 and 2020 stratified by Age** | | | | | | | | | | | | | | | | | |
| Year | 18-29 | | | | | 30-49 | | | | | | 50-64 | | | | | |
|  | Prevalence estimate | | Lower confidence limit | Upper confidence limit | | Prevalence estimate | | Lower confidence limit | Upper confidence limit | | | Prevalence estimate | | Lower confidence limit | | Upper confidence limit | |
| 1991 | 0.01 | | 0.01 | 0.01 | | 0.02 | | 0.02 | 0.03 | | | 0.04 | | 0.03 | | 0.04 | |
| 1992 | 0.01 | | 0.01 | 0.01 | | 0.03 | | 0.03 | 0.04 | | | 0.05 | | 0.05 | | 0.06 | |
| 1993 | 0.01 | | 0.01 | 0.01 | | 0.04 | | 0.04 | 0.04 | | | 0.07 | | 0.06 | | 0.07 | |
| 1994 | 0.01 | | 0.01 | 0.01 | | 0.05 | | 0.05 | 0.05 | | | 0.08 | | 0.08 | | 0.09 | |
| 1995 | 0.02 | | 0.01 | 0.02 | | 0.06 | | 0.06 | 0.07 | | | 0.10 | | 0.09 | | 0.11 | |
| 1996 | 0.02 | | 0.02 | 0.02 | | 0.07 | | 0.06 | 0.07 | | | 0.11 | | 0.10 | | 0.11 | |
| 1997 | 0.02 | | 0.02 | 0.02 | | 0.07 | | 0.07 | 0.07 | | | 0.12 | | 0.11 | | 0.12 | |
| 1998 | 0.02 | | 0.02 | 0.02 | | 0.08 | | 0.07 | 0.08 | | | 0.13 | | 0.12 | | 0.13 | |
| 1999 | 0.02 | | 0.02 | 0.02 | | 0.08 | | 0.07 | 0.08 | | | 0.13 | | 0.12 | | 0.14 | |
| 2000 | 0.02 | | 0.02 | 0.02 | | 0.08 | | 0.08 | 0.08 | | | 0.13 | | 0.13 | | 0.14 | |
| 2001 | 0.03 | | 0.02 | 0.03 | | 0.10 | | 0.10 | 0.10 | | | 0.16 | | 0.16 | | 0.17 | |
| 2002 | 0.03 | | 0.03 | 0.03 | | 0.11 | | 0.11 | 0.12 | | | 0.18 | | 0.18 | | 0.19 | |
| 2003 | 0.03 | | 0.03 | 0.03 | | 0.12 | | 0.12 | 0.13 | | | 0.20 | | 0.20 | | 0.21 | |
| 2004 | 0.03 | | 0.03 | 0.04 | | 0.14 | | 0.13 | 0.14 | | | 0.22 | | 0.22 | | 0.23 | |
| 2005 | 0.04 | | 0.04 | 0.04 | | 0.15 | | 0.15 | 0.16 | | | 0.25 | | 0.25 | | 0.26 | |
| 2006 | 0.04 | | 0.04 | 0.05 | | 0.17 | | 0.17 | 0.18 | | | 0.28 | | 0.28 | | 0.29 | |
| 2007 | 0.05 | | 0.05 | 0.05 | | 0.19 | | 0.18 | 0.19 | | | 0.31 | | 0.30 | | 0.31 | |
| 2008 | 0.05 | | 0.05 | 0.05 | | 0.20 | | 0.20 | 0.21 | | | 0.33 | | 0.32 | | 0.34 | |
| 2009 | 0.06 | | 0.05 | 0.06 | | 0.22 | | 0.21 | 0.22 | | | 0.36 | | 0.35 | | 0.37 | |
| 2010 | 0.06 | | 0.06 | 0.06 | | 0.23 | | 0.23 | 0.24 | | | 0.38 | | 0.38 | | 0.39 | |
| 2011 | 0.06 | | 0.06 | 0.07 | | 0.25 | | 0.25 | 0.25 | | | 0.41 | | 0.40 | | 0.42 | |
| 2012 | 0.07 | | 0.07 | 0.07 | | 0.27 | | 0.26 | 0.27 | | | 0.44 | | 0.43 | | 0.44 | |
| 2013 | 0.07 | | 0.07 | 0.07 | | 0.28 | | 0.27 | 0.28 | | | 0.46 | | 0.45 | | 0.47 | |
| 2014 | 0.07 | | 0.07 | 0.08 | | 0.29 | | 0.29 | 0.30 | | | 0.48 | | 0.47 | | 0.49 | |
| 2015 | 0.08 | | 0.07 | 0.08 | | 0.30 | | 0.30 | 0.31 | | | 0.49 | | 0.49 | | 0.50 | |
| 2016 | 0.08 | | 0.08 | 0.08 | | 0.31 | | 0.31 | 0.32 | | | 0.51 | | 0.50 | | 0.52 | |
| 2017 | 0.08 | | 0.08 | 0.08 | | 0.32 | | 0.31 | 0.33 | | | 0.52 | | 0.51 | | 0.53 | |
| 2018 | 0.08 | | 0.08 | 0.09 | | 0.33 | | 0.32 | 0.33 | | | 0.54 | | 0.53 | | 0.55 | |
| 2019 | 0.09 | | 0.08 | 0.09 | | 0.33 | | 0.33 | 0.34 | | | 0.55 | | 0.54 | | 0.56 | |
| 2020 | 0.08 | | 0.08 | 0.09 | | 0.33 | | 0.32 | 0.34 | | | 0.54 | | 0.53 | | 0.56 | |
|  |  | |  |  | |  | |  |  | | |  | |  | |  | |
| Year | 65-79 | | | | | 80+ | | | | | |  |  |  |  |  |  |
|  | Prevalence estimate | | Lower confidence limit | Upper confidence limit | | Prevalence estimate | | Lower confidence limit | Upper confidence limit | | |  |  |  |  |  |  |
| 1991 | 0.04 | | 0.03 | 0.04 | | 0.02 | | 0.02 | 0.03 | | |  |  |  |  |  |  |
| 1992 | 0.05 | | 0.05 | 0.06 | | 0.03 | | 0.03 | 0.04 | | |  |  |  |  |  |  |
| 1993 | 0.06 | | 0.06 | 0.07 | | 0.04 | | 0.04 | 0.04 | | |  |  |  |  |  |  |
| 1994 | 0.08 | | 0.07 | 0.08 | | 0.05 | | 0.05 | 0.05 | | |  |  |  |  |  |  |
| 1995 | 0.10 | | 0.09 | 0.10 | | 0.06 | | 0.06 | 0.07 | | |  |  |  |  |  |  |
| 1996 | 0.10 | | 0.09 | 0.11 | | 0.07 | | 0.06 | 0.07 | | |  |  |  |  |  |  |
| 1997 | 0.11 | | 0.10 | 0.12 | | 0.07 | | 0.07 | 0.07 | | |  |  |  |  |  |  |
| 1998 | 0.12 | | 0.11 | 0.12 | | 0.08 | | 0.07 | 0.08 | | |  |  |  |  |  |  |
| 1999 | 0.12 | | 0.12 | 0.13 | | 0.08 | | 0.07 | 0.08 | | |  |  |  |  |  |  |
| 2000 | 0.13 | | 0.12 | 0.13 | | 0.08 | | 0.08 | 0.09 | | |  |  |  |  |  |  |
| 2001 | 0.16 | | 0.15 | 0.16 | | 0.10 | | 0.10 | 0.10 | | |  |  |  |  |  |  |
| 2002 | 0.17 | | 0.17 | 0.18 | | 0.11 | | 0.11 | 0.12 | | |  |  |  |  |  |  |
| 2003 | 0.19 | | 0.19 | 0.20 | | 0.12 | | 0.12 | 0.13 | | |  |  |  |  |  |  |
| 2004 | 0.21 | | 0.21 | 0.22 | | 0.14 | | 0.13 | 0.14 | | |  |  |  |  |  |  |
| 2005 | 0.24 | | 0.23 | 0.24 | | 0.15 | | 0.15 | 0.16 | | |  |  |  |  |  |  |
| 2006 | 0.27 | | 0.26 | 0.27 | | 0.17 | | 0.17 | 0.18 | | |  |  |  |  |  |  |
| 2007 | 0.29 | | 0.28 | 0.30 | | 0.19 | | 0.18 | 0.19 | | |  |  |  |  |  |  |
| 2008 | 0.31 | | 0.31 | 0.32 | | 0.20 | | 0.20 | 0.21 | | |  |  |  |  |  |  |
| 2009 | 0.34 | | 0.33 | 0.35 | | 0.22 | | 0.21 | 0.22 | | |  |  |  |  |  |  |
| 2010 | 0.36 | | 0.36 | 0.37 | | 0.23 | | 0.23 | 0.24 | | |  |  |  |  |  |  |
| 2011 | 0.39 | | 0.38 | 0.40 | | 0.25 | | 0.25 | 0.26 | | |  |  |  |  |  |  |
| 2012 | 0.41 | | 0.41 | 0.42 | | 0.27 | | 0.26 | 0.27 | | |  |  |  |  |  |  |
| 2013 | 0.43 | | 0.43 | 0.44 | | 0.28 | | 0.27 | 0.29 | | |  |  |  |  |  |  |
| 2014 | 0.45 | | 0.44 | 0.46 | | 0.29 | | 0.29 | 0.30 | | |  |  |  |  |  |  |
| 2015 | 0.47 | | 0.46 | 0.48 | | 0.30 | | 0.29 | 0.31 | | |  |  |  |  |  |  |
| 2016 | 0.48 | | 0.47 | 0.49 | | 0.31 | | 0.30 | 0.32 | | |  |  |  |  |  |  |
| 2017 | 0.50 | | 0.49 | 0.51 | | 0.32 | | 0.31 | 0.33 | | |  |  |  |  |  |  |
| 2018 | 0.51 | | 0.50 | 0.52 | | 0.33 | | 0.32 | 0.34 | | |  |  |  |  |  |  |
| 2019 | 0.52 | | 0.51 | 0.53 | | 0.34 | | 0.33 | 0.34 | | |  |  |  |  |  |  |
| 2020 | 0.51 | | 0.50 | 0.53 | | 0.33 | | 0.32 | 0.34 | | |  |  |  |  |  |  |

| **Supplementary Table S6 – Annual estimates of PsA prevalence in the UK between 1991 and 2020 stratified by IMD decile (1=most deprived, 10=least deprived).** | | | | | | | | | |
| --- | --- | --- | --- | --- | --- | --- | --- | --- | --- |
| Year | 1 (most deprived) | | | 2 | | | 3 | | |
|  | Prevalence estimate | Lower confidence limit | Upper confidence limit | Prevalence estimate | Lower confidence limit | Upper confidence limit | Prevalence estimate | Lower confidence limit | Upper confidence limit |
| 1991 | 0.03 | 0.03 | 0.03 | 0.03 | 0.02 | 0.03 | 0.03 | 0.02 | 0.03 |
| 1992 | 0.04 | 0.04 | 0.04 | 0.04 | 0.03 | 0.04 | 0.04 | 0.03 | 0.04 |
| 1993 | 0.05 | 0.04 | 0.05 | 0.05 | 0.04 | 0.05 | 0.04 | 0.04 | 0.05 |
| 1994 | 0.06 | 0.06 | 0.07 | 0.06 | 0.05 | 0.06 | 0.05 | 0.05 | 0.06 |
| 1995 | 0.07 | 0.07 | 0.08 | 0.07 | 0.06 | 0.07 | 0.07 | 0.06 | 0.07 |
| 1996 | 0.08 | 0.07 | 0.08 | 0.07 | 0.07 | 0.08 | 0.07 | 0.07 | 0.08 |
| 1997 | 0.09 | 0.08 | 0.09 | 0.08 | 0.08 | 0.08 | 0.08 | 0.07 | 0.08 |
| 1998 | 0.09 | 0.09 | 0.10 | 0.09 | 0.08 | 0.09 | 0.08 | 0.08 | 0.09 |
| 1999 | 0.10 | 0.09 | 0.10 | 0.09 | 0.08 | 0.09 | 0.09 | 0.08 | 0.09 |
| 2000 | 0.10 | 0.10 | 0.10 | 0.09 | 0.09 | 0.10 | 0.09 | 0.09 | 0.09 |
| 2001 | 0.12 | 0.12 | 0.13 | 0.11 | 0.11 | 0.12 | 0.11 | 0.11 | 0.11 |
| 2002 | 0.14 | 0.13 | 0.14 | 0.13 | 0.12 | 0.13 | 0.12 | 0.12 | 0.13 |
| 2003 | 0.15 | 0.15 | 0.16 | 0.14 | 0.14 | 0.14 | 0.14 | 0.13 | 0.14 |
| 2004 | 0.16 | 0.16 | 0.17 | 0.15 | 0.15 | 0.16 | 0.15 | 0.14 | 0.15 |
| 2005 | 0.19 | 0.18 | 0.19 | 0.17 | 0.17 | 0.18 | 0.17 | 0.16 | 0.17 |
| 2006 | 0.21 | 0.20 | 0.21 | 0.19 | 0.19 | 0.20 | 0.19 | 0.18 | 0.19 |
| 2007 | 0.23 | 0.22 | 0.23 | 0.21 | 0.20 | 0.21 | 0.20 | 0.20 | 0.21 |
| 2008 | 0.24 | 0.24 | 0.25 | 0.23 | 0.22 | 0.23 | 0.22 | 0.22 | 0.23 |
| 2009 | 0.26 | 0.26 | 0.27 | 0.25 | 0.24 | 0.25 | 0.24 | 0.23 | 0.24 |
| 2010 | 0.28 | 0.28 | 0.29 | 0.26 | 0.26 | 0.27 | 0.26 | 0.25 | 0.26 |
| 2011 | 0.30 | 0.30 | 0.31 | 0.28 | 0.27 | 0.29 | 0.27 | 0.27 | 0.28 |
| 2012 | 0.31 | 0.31 | 0.32 | 0.30 | 0.29 | 0.30 | 0.29 | 0.28 | 0.30 |
| 2013 | 0.33 | 0.32 | 0.34 | 0.31 | 0.31 | 0.32 | 0.31 | 0.30 | 0.31 |
| 2014 | 0.34 | 0.34 | 0.35 | 0.33 | 0.32 | 0.33 | 0.32 | 0.31 | 0.32 |
| 2015 | 0.36 | 0.35 | 0.36 | 0.34 | 0.33 | 0.34 | 0.33 | 0.32 | 0.34 |
| 2016 | 0.37 | 0.36 | 0.38 | 0.35 | 0.34 | 0.36 | 0.34 | 0.33 | 0.35 |
| 2017 | 0.38 | 0.37 | 0.39 | 0.36 | 0.35 | 0.37 | 0.35 | 0.34 | 0.36 |
| 2018 | 0.39 | 0.38 | 0.40 | 0.37 | 0.36 | 0.37 | 0.36 | 0.35 | 0.37 |
| 2019 | 0.40 | 0.39 | 0.40 | 0.37 | 0.37 | 0.38 | 0.37 | 0.36 | 0.37 |
| 2020 | 0.39 | 0.38 | 0.40 | 0.37 | 0.36 | 0.38 | 0.36 | 0.35 | 0.37 |
|  |  |  |  |  |  |  |  |  |  |
| Year | 4 | | | 5 | | | 6 | | |
|  | Prevalence estimate | Lower confidence limit | Upper confidence limit | Prevalence estimate | Lower confidence limit | Upper confidence limit | Prevalence estimate | Lower confidence limit | Upper confidence limit |
| 1991 | 0.03 | 0.02 | 0.03 | 0.03 | 0.02 | 0.03 | 0.03 | 0.02 | 0.03 |
| 1992 | 0.04 | 0.03 | 0.04 | 0.03 | 0.03 | 0.04 | 0.03 | 0.03 | 0.04 |
| 1993 | 0.04 | 0.04 | 0.05 | 0.04 | 0.04 | 0.05 | 0.04 | 0.04 | 0.05 |
| 1994 | 0.05 | 0.05 | 0.06 | 0.05 | 0.05 | 0.06 | 0.05 | 0.05 | 0.06 |
| 1995 | 0.07 | 0.06 | 0.07 | 0.06 | 0.06 | 0.07 | 0.06 | 0.06 | 0.07 |
| 1996 | 0.07 | 0.07 | 0.07 | 0.07 | 0.06 | 0.07 | 0.07 | 0.06 | 0.07 |
| 1997 | 0.08 | 0.07 | 0.08 | 0.07 | 0.07 | 0.08 | 0.07 | 0.07 | 0.08 |
| 1998 | 0.08 | 0.08 | 0.09 | 0.08 | 0.08 | 0.08 | 0.08 | 0.08 | 0.08 |
| 1999 | 0.08 | 0.08 | 0.09 | 0.08 | 0.08 | 0.09 | 0.08 | 0.08 | 0.09 |
| 2000 | 0.09 | 0.08 | 0.09 | 0.09 | 0.08 | 0.09 | 0.09 | 0.08 | 0.09 |
| 2001 | 0.11 | 0.10 | 0.11 | 0.11 | 0.10 | 0.11 | 0.11 | 0.10 | 0.11 |
| 2002 | 0.12 | 0.12 | 0.12 | 0.12 | 0.11 | 0.12 | 0.12 | 0.11 | 0.12 |
| 2003 | 0.13 | 0.13 | 0.14 | 0.13 | 0.13 | 0.13 | 0.13 | 0.13 | 0.14 |
| 2004 | 0.15 | 0.14 | 0.15 | 0.14 | 0.14 | 0.15 | 0.14 | 0.14 | 0.15 |
| 2005 | 0.17 | 0.16 | 0.17 | 0.16 | 0.16 | 0.17 | 0.16 | 0.16 | 0.17 |
| 2006 | 0.18 | 0.18 | 0.19 | 0.18 | 0.18 | 0.19 | 0.18 | 0.18 | 0.18 |
| 2007 | 0.20 | 0.20 | 0.21 | 0.20 | 0.19 | 0.20 | 0.20 | 0.19 | 0.20 |
| 2008 | 0.22 | 0.21 | 0.22 | 0.21 | 0.21 | 0.22 | 0.21 | 0.21 | 0.22 |
| 2009 | 0.24 | 0.23 | 0.24 | 0.23 | 0.23 | 0.24 | 0.23 | 0.23 | 0.24 |
| 2010 | 0.25 | 0.25 | 0.26 | 0.25 | 0.24 | 0.25 | 0.25 | 0.24 | 0.25 |
| 2011 | 0.27 | 0.26 | 0.28 | 0.26 | 0.26 | 0.27 | 0.26 | 0.26 | 0.27 |
| 2012 | 0.29 | 0.28 | 0.29 | 0.28 | 0.27 | 0.29 | 0.28 | 0.27 | 0.29 |
| 2013 | 0.30 | 0.29 | 0.31 | 0.29 | 0.29 | 0.30 | 0.29 | 0.29 | 0.30 |
| 2014 | 0.31 | 0.31 | 0.32 | 0.31 | 0.30 | 0.31 | 0.31 | 0.30 | 0.31 |
| 2015 | 0.32 | 0.32 | 0.33 | 0.32 | 0.31 | 0.32 | 0.32 | 0.31 | 0.32 |
| 2016 | 0.34 | 0.33 | 0.34 | 0.33 | 0.32 | 0.34 | 0.33 | 0.32 | 0.34 |
| 2017 | 0.34 | 0.34 | 0.35 | 0.34 | 0.33 | 0.34 | 0.34 | 0.33 | 0.34 |
| 2018 | 0.35 | 0.34 | 0.36 | 0.34 | 0.34 | 0.35 | 0.34 | 0.34 | 0.35 |
| 2019 | 0.36 | 0.35 | 0.37 | 0.35 | 0.34 | 0.36 | 0.35 | 0.34 | 0.36 |
| 2020 | 0.36 | 0.35 | 0.36 | 0.35 | 0.34 | 0.36 | 0.35 | 0.34 | 0.36 |
|  |  |  |  |  |  |  |  |  |  |
| Year | 7 | | | 8 | | | 9 | | |
|  | Prevalence estimate | Lower confidence limit | Upper confidence limit | Prevalence estimate | Lower confidence limit | Upper confidence limit | Prevalence estimate | Lower confidence limit | Upper confidence limit |
| 1991 | 0.03 | 0.02 | 0.03 | 0.02 | 0.02 | 0.03 | 0.03 | 0.03 | 0.03 |
| 1992 | 0.04 | 0.03 | 0.04 | 0.03 | 0.03 | 0.04 | 0.04 | 0.04 | 0.04 |
| 1993 | 0.04 | 0.04 | 0.05 | 0.04 | 0.04 | 0.05 | 0.05 | 0.04 | 0.05 |
| 1994 | 0.05 | 0.05 | 0.06 | 0.05 | 0.05 | 0.06 | 0.06 | 0.06 | 0.07 |
| 1995 | 0.07 | 0.06 | 0.07 | 0.06 | 0.06 | 0.07 | 0.07 | 0.07 | 0.08 |
| 1996 | 0.07 | 0.07 | 0.07 | 0.07 | 0.06 | 0.07 | 0.08 | 0.07 | 0.08 |
| 1997 | 0.08 | 0.07 | 0.08 | 0.07 | 0.07 | 0.08 | 0.09 | 0.08 | 0.09 |
| 1998 | 0.08 | 0.08 | 0.09 | 0.08 | 0.08 | 0.08 | 0.09 | 0.09 | 0.10 |
| 1999 | 0.09 | 0.08 | 0.09 | 0.08 | 0.08 | 0.09 | 0.10 | 0.09 | 0.10 |
| 2000 | 0.09 | 0.08 | 0.09 | 0.08 | 0.08 | 0.09 | 0.10 | 0.10 | 0.10 |
| 2001 | 0.11 | 0.10 | 0.11 | 0.10 | 0.10 | 0.11 | 0.12 | 0.12 | 0.13 |
| 2002 | 0.12 | 0.12 | 0.12 | 0.12 | 0.11 | 0.12 | 0.14 | 0.13 | 0.14 |
| 2003 | 0.13 | 0.13 | 0.14 | 0.13 | 0.13 | 0.13 | 0.15 | 0.15 | 0.16 |
| 2004 | 0.15 | 0.14 | 0.15 | 0.14 | 0.14 | 0.14 | 0.17 | 0.16 | 0.17 |
| 2005 | 0.17 | 0.16 | 0.17 | 0.16 | 0.15 | 0.16 | 0.19 | 0.18 | 0.19 |
| 2006 | 0.19 | 0.18 | 0.19 | 0.18 | 0.17 | 0.18 | 0.21 | 0.20 | 0.21 |
| 2007 | 0.20 | 0.20 | 0.21 | 0.19 | 0.19 | 0.20 | 0.23 | 0.22 | 0.23 |
| 2008 | 0.22 | 0.21 | 0.22 | 0.21 | 0.20 | 0.21 | 0.25 | 0.24 | 0.25 |
| 2009 | 0.24 | 0.23 | 0.24 | 0.23 | 0.22 | 0.23 | 0.27 | 0.26 | 0.27 |
| 2010 | 0.25 | 0.25 | 0.26 | 0.24 | 0.24 | 0.25 | 0.29 | 0.28 | 0.29 |
| 2011 | 0.27 | 0.26 | 0.28 | 0.26 | 0.25 | 0.26 | 0.31 | 0.30 | 0.31 |
| 2012 | 0.29 | 0.28 | 0.29 | 0.28 | 0.27 | 0.28 | 0.32 | 0.32 | 0.33 |
| 2013 | 0.30 | 0.30 | 0.31 | 0.29 | 0.28 | 0.30 | 0.34 | 0.33 | 0.35 |
| 2014 | 0.31 | 0.31 | 0.32 | 0.30 | 0.29 | 0.31 | 0.35 | 0.35 | 0.36 |
| 2015 | 0.33 | 0.32 | 0.33 | 0.31 | 0.30 | 0.32 | 0.37 | 0.36 | 0.37 |
| 2016 | 0.34 | 0.33 | 0.34 | 0.32 | 0.32 | 0.33 | 0.38 | 0.37 | 0.39 |
| 2017 | 0.34 | 0.34 | 0.35 | 0.33 | 0.32 | 0.34 | 0.39 | 0.38 | 0.40 |
| 2018 | 0.35 | 0.34 | 0.36 | 0.34 | 0.33 | 0.35 | 0.40 | 0.39 | 0.41 |
| 2019 | 0.36 | 0.35 | 0.37 | 0.35 | 0.34 | 0.35 | 0.41 | 0.40 | 0.42 |
| 2020 | 0.36 | 0.35 | 0.37 | 0.34 | 0.33 | 0.35 | 0.40 | 0.39 | 0.41 |
|  |  |  |  |  |  |  |  |  |  |
| Year | 10 (least deprived) | | |  |  |  |  |  |  |
|  | Prevalence estimate | Lower confidence limit | Upper confidence limit |  |  |  |  |  |  |
| 1991 | 0.03 | 0.03 | 0.03 |  |  |  |  |  |  |
| 1992 | 0.04 | 0.04 | 0.04 |  |  |  |  |  |  |
| 1993 | 0.05 | 0.04 | 0.05 |  |  |  |  |  |  |
| 1994 | 0.06 | 0.06 | 0.07 |  |  |  |  |  |  |
| 1995 | 0.07 | 0.07 | 0.08 |  |  |  |  |  |  |
| 1996 | 0.08 | 0.07 | 0.08 |  |  |  |  |  |  |
| 1997 | 0.09 | 0.08 | 0.09 |  |  |  |  |  |  |
| 1998 | 0.09 | 0.09 | 0.10 |  |  |  |  |  |  |
| 1999 | 0.10 | 0.09 | 0.10 |  |  |  |  |  |  |
| 2000 | 0.10 | 0.10 | 0.10 |  |  |  |  |  |  |
| 2001 | 0.12 | 0.12 | 0.13 |  |  |  |  |  |  |
| 2002 | 0.14 | 0.13 | 0.14 |  |  |  |  |  |  |
| 2003 | 0.15 | 0.15 | 0.16 |  |  |  |  |  |  |
| 2004 | 0.16 | 0.16 | 0.17 |  |  |  |  |  |  |
| 2005 | 0.19 | 0.18 | 0.19 |  |  |  |  |  |  |
| 2006 | 0.21 | 0.20 | 0.21 |  |  |  |  |  |  |
| 2007 | 0.23 | 0.22 | 0.23 |  |  |  |  |  |  |
| 2008 | 0.24 | 0.24 | 0.25 |  |  |  |  |  |  |
| 2009 | 0.26 | 0.26 | 0.27 |  |  |  |  |  |  |
| 2010 | 0.28 | 0.28 | 0.29 |  |  |  |  |  |  |
| 2011 | 0.30 | 0.30 | 0.31 |  |  |  |  |  |  |
| 2012 | 0.32 | 0.32 | 0.33 |  |  |  |  |  |  |
| 2013 | 0.34 | 0.33 | 0.35 |  |  |  |  |  |  |
| 2014 | 0.35 | 0.34 | 0.36 |  |  |  |  |  |  |
| 2015 | 0.37 | 0.36 | 0.37 |  |  |  |  |  |  |
| 2016 | 0.38 | 0.37 | 0.39 |  |  |  |  |  |  |
| 2017 | 0.39 | 0.38 | 0.40 |  |  |  |  |  |  |
| 2018 | 0.40 | 0.39 | 0.41 |  |  |  |  |  |  |
| 2019 | 0.41 | 0.40 | 0.42 |  |  |  |  |  |  |
| 2020 | 0.40 | 0.39 | 0.41 |  |  |  |  |  |  |

| **Supplementary Table S7 – Annual estimates of PsA prevalence in the UK between 1991 and 2020 stratified by Region** | | | | | | | | | |
| --- | --- | --- | --- | --- | --- | --- | --- | --- | --- |
| Year | East midlands | | | East of England | | | London | | |
|  | Prevalence estimate | Lower confidence limit | Upper confidence limit | Prevalence estimate | Lower confidence limit | Upper confidence limit | Prevalence estimate | Lower confidence limit | Upper confidence limit |
| 1991 | 0.03 | 0.03 | 0.03 | 0.02 | 0.02 | 0.03 | 0.02 | 0.02 | 0.02 |
| 1992 | 0.04 | 0.04 | 0.04 | 0.03 | 0.03 | 0.04 | 0.02 | 0.02 | 0.03 |
| 1993 | 0.05 | 0.04 | 0.05 | 0.04 | 0.04 | 0.05 | 0.03 | 0.03 | 0.03 |
| 1994 | 0.06 | 0.06 | 0.07 | 0.05 | 0.05 | 0.06 | 0.04 | 0.03 | 0.04 |
| 1995 | 0.08 | 0.07 | 0.08 | 0.06 | 0.06 | 0.07 | 0.05 | 0.04 | 0.05 |
| 1996 | 0.08 | 0.08 | 0.09 | 0.07 | 0.06 | 0.07 | 0.05 | 0.05 | 0.05 |
| 1997 | 0.09 | 0.08 | 0.09 | 0.07 | 0.07 | 0.08 | 0.05 | 0.05 | 0.06 |
| 1998 | 0.09 | 0.09 | 0.10 | 0.08 | 0.07 | 0.08 | 0.06 | 0.05 | 0.06 |
| 1999 | 0.10 | 0.09 | 0.10 | 0.08 | 0.08 | 0.08 | 0.06 | 0.06 | 0.06 |
| 2000 | 0.10 | 0.10 | 0.10 | 0.08 | 0.08 | 0.09 | 0.06 | 0.06 | 0.06 |
| 2001 | 0.12 | 0.12 | 0.13 | 0.10 | 0.10 | 0.11 | 0.07 | 0.07 | 0.08 |
| 2002 | 0.14 | 0.13 | 0.14 | 0.12 | 0.11 | 0.12 | 0.08 | 0.08 | 0.09 |
| 2003 | 0.15 | 0.15 | 0.16 | 0.13 | 0.12 | 0.13 | 0.09 | 0.09 | 0.10 |
| 2004 | 0.17 | 0.16 | 0.17 | 0.14 | 0.13 | 0.14 | 0.10 | 0.10 | 0.10 |
| 2005 | 0.19 | 0.18 | 0.19 | 0.16 | 0.15 | 0.16 | 0.11 | 0.11 | 0.12 |
| 2006 | 0.21 | 0.20 | 0.22 | 0.18 | 0.17 | 0.18 | 0.13 | 0.12 | 0.13 |
| 2007 | 0.23 | 0.22 | 0.24 | 0.19 | 0.19 | 0.20 | 0.14 | 0.13 | 0.14 |
| 2008 | 0.25 | 0.24 | 0.25 | 0.21 | 0.20 | 0.21 | 0.15 | 0.14 | 0.15 |
| 2009 | 0.27 | 0.26 | 0.28 | 0.22 | 0.22 | 0.23 | 0.16 | 0.16 | 0.17 |
| 2010 | 0.29 | 0.28 | 0.30 | 0.24 | 0.24 | 0.25 | 0.17 | 0.17 | 0.18 |
| 2011 | 0.31 | 0.30 | 0.32 | 0.26 | 0.25 | 0.26 | 0.18 | 0.18 | 0.19 |
| 2012 | 0.33 | 0.31 | 0.34 | 0.27 | 0.27 | 0.28 | 0.20 | 0.19 | 0.20 |
| 2013 | 0.34 | 0.33 | 0.35 | 0.29 | 0.28 | 0.29 | 0.21 | 0.20 | 0.21 |
| 2014 | 0.36 | 0.34 | 0.37 | 0.30 | 0.29 | 0.31 | 0.21 | 0.21 | 0.22 |
| 2015 | 0.37 | 0.36 | 0.38 | 0.31 | 0.30 | 0.32 | 0.22 | 0.22 | 0.23 |
| 2016 | 0.38 | 0.37 | 0.39 | 0.32 | 0.31 | 0.33 | 0.23 | 0.22 | 0.24 |
| 2017 | 0.39 | 0.38 | 0.40 | 0.33 | 0.32 | 0.34 | 0.24 | 0.23 | 0.24 |
| 2018 | 0.40 | 0.39 | 0.41 | 0.34 | 0.33 | 0.34 | 0.24 | 0.24 | 0.25 |
| 2019 | 0.41 | 0.39 | 0.42 | 0.34 | 0.33 | 0.35 | 0.25 | 0.24 | 0.25 |
| 2020 | 0.40 | 0.39 | 0.42 | 0.34 | 0.33 | 0.35 | 0.24 | 0.24 | 0.25 |
|  |  |  |  |  |  |  |  |  |  |
| Year | North East | | | North West | | | Northern Ireland | | |
|  | Prevalence estimate | Lower confidence limit | Upper confidence limit | Prevalence estimate | Lower confidence limit | Upper confidence limit | Prevalence estimate | Lower confidence limit | Upper confidence limit |
| 1991 | 0.03 | 0.03 | 0.04 | 0.03 | 0.03 | 0.04 | 0.04 | 0.03 | 0.04 |
| 1992 | 0.05 | 0.04 | 0.05 | 0.04 | 0.04 | 0.05 | 0.05 | 0.05 | 0.06 |
| 1993 | 0.06 | 0.05 | 0.06 | 0.05 | 0.05 | 0.06 | 0.06 | 0.06 | 0.07 |
| 1994 | 0.07 | 0.06 | 0.08 | 0.07 | 0.06 | 0.07 | 0.08 | 0.07 | 0.09 |
| 1995 | 0.09 | 0.08 | 0.09 | 0.08 | 0.08 | 0.09 | 0.10 | 0.09 | 0.10 |
| 1996 | 0.09 | 0.09 | 0.10 | 0.09 | 0.08 | 0.09 | 0.10 | 0.10 | 0.11 |
| 1997 | 0.10 | 0.09 | 0.11 | 0.09 | 0.09 | 0.10 | 0.11 | 0.11 | 0.12 |
| 1998 | 0.11 | 0.10 | 0.11 | 0.10 | 0.10 | 0.11 | 0.12 | 0.11 | 0.13 |
| 1999 | 0.11 | 0.10 | 0.12 | 0.11 | 0.10 | 0.11 | 0.12 | 0.12 | 0.13 |
| 2000 | 0.12 | 0.11 | 0.12 | 0.11 | 0.11 | 0.11 | 0.13 | 0.12 | 0.13 |
| 2001 | 0.14 | 0.13 | 0.15 | 0.13 | 0.13 | 0.14 | 0.16 | 0.15 | 0.16 |
| 2002 | 0.16 | 0.15 | 0.17 | 0.15 | 0.14 | 0.15 | 0.18 | 0.17 | 0.18 |
| 2003 | 0.18 | 0.17 | 0.18 | 0.17 | 0.16 | 0.17 | 0.20 | 0.19 | 0.20 |
| 2004 | 0.19 | 0.18 | 0.20 | 0.18 | 0.18 | 0.19 | 0.21 | 0.21 | 0.22 |
| 2005 | 0.22 | 0.21 | 0.23 | 0.20 | 0.20 | 0.21 | 0.24 | 0.24 | 0.25 |
| 2006 | 0.24 | 0.23 | 0.25 | 0.23 | 0.22 | 0.24 | 0.27 | 0.26 | 0.28 |
| 2007 | 0.26 | 0.25 | 0.27 | 0.25 | 0.24 | 0.26 | 0.29 | 0.29 | 0.30 |
| 2008 | 0.28 | 0.27 | 0.30 | 0.27 | 0.26 | 0.27 | 0.32 | 0.31 | 0.33 |
| 2009 | 0.31 | 0.30 | 0.32 | 0.29 | 0.29 | 0.30 | 0.35 | 0.34 | 0.35 |
| 2010 | 0.33 | 0.32 | 0.34 | 0.31 | 0.31 | 0.32 | 0.37 | 0.36 | 0.38 |
| 2011 | 0.35 | 0.34 | 0.37 | 0.33 | 0.33 | 0.34 | 0.40 | 0.39 | 0.40 |
| 2012 | 0.37 | 0.36 | 0.39 | 0.36 | 0.35 | 0.36 | 0.42 | 0.41 | 0.43 |
| 2013 | 0.39 | 0.38 | 0.41 | 0.37 | 0.37 | 0.38 | 0.44 | 0.43 | 0.45 |
| 2014 | 0.41 | 0.39 | 0.43 | 0.39 | 0.38 | 0.40 | 0.46 | 0.45 | 0.47 |
| 2015 | 0.42 | 0.41 | 0.44 | 0.40 | 0.39 | 0.41 | 0.48 | 0.47 | 0.49 |
| 2016 | 0.44 | 0.42 | 0.46 | 0.42 | 0.41 | 0.43 | 0.49 | 0.48 | 0.50 |
| 2017 | 0.45 | 0.43 | 0.47 | 0.43 | 0.42 | 0.44 | 0.50 | 0.49 | 0.52 |
| 2018 | 0.46 | 0.44 | 0.48 | 0.44 | 0.43 | 0.45 | 0.52 | 0.50 | 0.53 |
| 2019 | 0.47 | 0.45 | 0.49 | 0.45 | 0.44 | 0.46 | 0.53 | 0.52 | 0.54 |
| 2020 | 0.47 | 0.45 | 0.49 | 0.44 | 0.43 | 0.45 | 0.52 | 0.51 | 0.53 |
|  |  |  |  |  |  |  |  |  |  |
| Year | Scotland | | | South East | | | South West | | |
|  | Prevalence estimate | Lower confidence limit | Upper confidence limit | Prevalence estimate | Lower confidence limit | Upper confidence limit | Prevalence estimate | Lower confidence limit | Upper confidence limit |
| 1991 | 0.02 | 0.02 | 0.03 | 0.03 | 0.02 | 0.03 | 0.03 | 0.02 | 0.03 |
| 1992 | 0.03 | 0.03 | 0.04 | 0.03 | 0.03 | 0.04 | 0.04 | 0.03 | 0.04 |
| 1993 | 0.04 | 0.04 | 0.04 | 0.04 | 0.04 | 0.05 | 0.05 | 0.04 | 0.05 |
| 1994 | 0.05 | 0.05 | 0.05 | 0.05 | 0.05 | 0.06 | 0.06 | 0.05 | 0.06 |
| 1995 | 0.06 | 0.06 | 0.07 | 0.06 | 0.06 | 0.07 | 0.07 | 0.07 | 0.08 |
| 1996 | 0.07 | 0.06 | 0.07 | 0.07 | 0.06 | 0.07 | 0.08 | 0.07 | 0.08 |
| 1997 | 0.07 | 0.07 | 0.07 | 0.07 | 0.07 | 0.08 | 0.08 | 0.08 | 0.09 |
| 1998 | 0.08 | 0.07 | 0.08 | 0.08 | 0.08 | 0.08 | 0.09 | 0.09 | 0.10 |
| 1999 | 0.08 | 0.08 | 0.08 | 0.08 | 0.08 | 0.09 | 0.09 | 0.09 | 0.10 |
| 2000 | 0.08 | 0.08 | 0.08 | 0.09 | 0.08 | 0.09 | 0.10 | 0.09 | 0.10 |
| 2001 | 0.10 | 0.10 | 0.10 | 0.10 | 0.10 | 0.11 | 0.12 | 0.11 | 0.12 |
| 2002 | 0.11 | 0.11 | 0.12 | 0.12 | 0.11 | 0.12 | 0.13 | 0.13 | 0.14 |
| 2003 | 0.12 | 0.12 | 0.13 | 0.13 | 0.13 | 0.13 | 0.15 | 0.14 | 0.15 |
| 2004 | 0.14 | 0.13 | 0.14 | 0.14 | 0.14 | 0.14 | 0.16 | 0.16 | 0.17 |
| 2005 | 0.15 | 0.15 | 0.16 | 0.16 | 0.16 | 0.16 | 0.18 | 0.18 | 0.19 |
| 2006 | 0.17 | 0.17 | 0.18 | 0.18 | 0.18 | 0.18 | 0.20 | 0.20 | 0.21 |
| 2007 | 0.19 | 0.18 | 0.19 | 0.19 | 0.19 | 0.20 | 0.22 | 0.22 | 0.23 |
| 2008 | 0.20 | 0.20 | 0.21 | 0.21 | 0.21 | 0.21 | 0.24 | 0.23 | 0.24 |
| 2009 | 0.22 | 0.21 | 0.22 | 0.23 | 0.22 | 0.23 | 0.26 | 0.25 | 0.27 |
| 2010 | 0.23 | 0.23 | 0.24 | 0.24 | 0.24 | 0.25 | 0.28 | 0.27 | 0.28 |
| 2011 | 0.25 | 0.25 | 0.26 | 0.26 | 0.26 | 0.27 | 0.30 | 0.29 | 0.30 |
| 2012 | 0.27 | 0.26 | 0.27 | 0.28 | 0.27 | 0.28 | 0.32 | 0.31 | 0.32 |
| 2013 | 0.28 | 0.28 | 0.28 | 0.29 | 0.29 | 0.30 | 0.33 | 0.32 | 0.34 |
| 2014 | 0.29 | 0.29 | 0.30 | 0.30 | 0.30 | 0.31 | 0.34 | 0.34 | 0.35 |
| 2015 | 0.30 | 0.30 | 0.31 | 0.31 | 0.31 | 0.32 | 0.36 | 0.35 | 0.37 |
| 2016 | 0.31 | 0.31 | 0.32 | 0.32 | 0.32 | 0.33 | 0.37 | 0.36 | 0.38 |
| 2017 | 0.32 | 0.31 | 0.33 | 0.33 | 0.33 | 0.34 | 0.38 | 0.37 | 0.39 |
| 2018 | 0.33 | 0.32 | 0.33 | 0.34 | 0.33 | 0.35 | 0.39 | 0.38 | 0.40 |
| 2019 | 0.33 | 0.33 | 0.34 | 0.35 | 0.34 | 0.36 | 0.40 | 0.39 | 0.41 |
| 2020 | 0.33 | 0.32 | 0.34 | 0.34 | 0.34 | 0.35 | 0.39 | 0.38 | 0.40 |
|  |  |  |  |  |  |  |  |  |  |
| Year | Wales | | | West Midlands | | | Yorkshire and The Humber | | |
|  | Prevalence estimate | Lower confidence limit | Upper confidence limit | Prevalence estimate | Lower confidence limit | Upper confidence limit | Prevalence estimate | Lower confidence limit | Upper confidence limit |
| 1991 | 0.03 | 0.02 | 0.03 | 0.03 | 0.02 | 0.03 | 0.03 | 0.03 | 0.04 |
| 1992 | 0.03 | 0.03 | 0.04 | 0.04 | 0.03 | 0.04 | 0.04 | 0.04 | 0.05 |
| 1993 | 0.04 | 0.04 | 0.05 | 0.04 | 0.04 | 0.05 | 0.05 | 0.05 | 0.06 |
| 1994 | 0.05 | 0.05 | 0.06 | 0.05 | 0.05 | 0.06 | 0.07 | 0.06 | 0.07 |
| 1995 | 0.06 | 0.06 | 0.07 | 0.07 | 0.06 | 0.07 | 0.08 | 0.08 | 0.09 |
| 1996 | 0.07 | 0.06 | 0.07 | 0.07 | 0.07 | 0.08 | 0.09 | 0.08 | 0.09 |
| 1997 | 0.07 | 0.07 | 0.08 | 0.08 | 0.07 | 0.08 | 0.09 | 0.09 | 0.10 |
| 1998 | 0.08 | 0.08 | 0.08 | 0.08 | 0.08 | 0.09 | 0.10 | 0.10 | 0.11 |
| 1999 | 0.08 | 0.08 | 0.09 | 0.09 | 0.08 | 0.09 | 0.11 | 0.10 | 0.11 |
| 2000 | 0.09 | 0.08 | 0.09 | 0.09 | 0.09 | 0.09 | 0.11 | 0.10 | 0.11 |
| 2001 | 0.10 | 0.10 | 0.11 | 0.11 | 0.11 | 0.11 | 0.13 | 0.13 | 0.14 |
| 2002 | 0.12 | 0.11 | 0.12 | 0.12 | 0.12 | 0.13 | 0.15 | 0.14 | 0.16 |
| 2003 | 0.13 | 0.13 | 0.13 | 0.14 | 0.13 | 0.14 | 0.17 | 0.16 | 0.17 |
| 2004 | 0.14 | 0.14 | 0.15 | 0.15 | 0.15 | 0.15 | 0.18 | 0.17 | 0.19 |
| 2005 | 0.16 | 0.16 | 0.16 | 0.17 | 0.16 | 0.17 | 0.20 | 0.20 | 0.21 |
| 2006 | 0.18 | 0.18 | 0.18 | 0.19 | 0.18 | 0.19 | 0.23 | 0.22 | 0.24 |
| 2007 | 0.20 | 0.19 | 0.20 | 0.20 | 0.20 | 0.21 | 0.25 | 0.24 | 0.26 |
| 2008 | 0.21 | 0.21 | 0.22 | 0.22 | 0.22 | 0.23 | 0.27 | 0.26 | 0.28 |
| 2009 | 0.23 | 0.23 | 0.23 | 0.24 | 0.23 | 0.25 | 0.29 | 0.28 | 0.30 |
| 2010 | 0.25 | 0.24 | 0.25 | 0.26 | 0.25 | 0.26 | 0.31 | 0.30 | 0.32 |
| 2011 | 0.26 | 0.26 | 0.27 | 0.28 | 0.27 | 0.28 | 0.33 | 0.32 | 0.35 |
| 2012 | 0.28 | 0.27 | 0.28 | 0.29 | 0.29 | 0.30 | 0.36 | 0.34 | 0.37 |
| 2013 | 0.29 | 0.29 | 0.30 | 0.31 | 0.30 | 0.31 | 0.37 | 0.36 | 0.39 |
| 2014 | 0.31 | 0.30 | 0.31 | 0.32 | 0.31 | 0.33 | 0.39 | 0.38 | 0.40 |
| 2015 | 0.32 | 0.31 | 0.32 | 0.33 | 0.32 | 0.34 | 0.40 | 0.39 | 0.42 |
| 2016 | 0.33 | 0.32 | 0.33 | 0.34 | 0.34 | 0.35 | 0.42 | 0.40 | 0.43 |
| 2017 | 0.33 | 0.33 | 0.34 | 0.35 | 0.34 | 0.36 | 0.43 | 0.41 | 0.44 |
| 2018 | 0.34 | 0.34 | 0.35 | 0.36 | 0.35 | 0.37 | 0.44 | 0.42 | 0.45 |
| 2019 | 0.35 | 0.34 | 0.36 | 0.37 | 0.36 | 0.38 | 0.45 | 0.43 | 0.46 |
| 2020 | 0.35 | 0.34 | 0.36 | 0.36 | 0.35 | 0.37 | 0.44 | 0.43 | 0.46 |

# Observed incidence in CPRD in 1991 and 2020, all cases and stratified by case definition, sex, age, deprivation and region

| **Supplementary Table S8 - Observed incidence of PsA in CPRD in 1991** | | | |
| --- | --- | --- | --- |
| Stratification variable | Cases | Person time (years) | Incidence rate |
| **Case definition** | | | |
| All | 67 | 757119 | 8.85 |
| Definition 1: Definite PsA | 49 | 757119 | 6.47 |
| Definition 2: Probable PsA* | 18 | 757119 | 2.38 |
| Definition 3: Probable PsA** | 0 | 757119 | 0.00 |
| **Stratified by sex** | | | |
| Male | 37 | 364425 | 8.23 |
| Female | 30 | 392694 | 9.42 |
| **Stratified by age** | | | |
| 18-29 | 6 | 159187 | 3.77 |
| 30-49 | 28 | 276588 | 10.12 |
| 50-64 | 21 | 153949 | 13.64 |
| 65-79 | 12 | 125085 | 9.59 |
| 80+ | 0 | 42310 | 0.00 |
| **Stratified by IMD decile (1=most deprived, 10=least deprived).** | | | |
| 1 | 6 | 64800 | 9.26 |
| 2 | 1 | 34717 | 2.88 |
| 3 | 11 | 69797 | 15.76 |
| 4 | 6 | 47751 | 12.57 |
| 5 | 2 | 57941 | 3.45 |
| 6 | 2 | 88551 | 2.26 |
| 7 | 10 | 85736 | 11.66 |
| 8 | 9 | 95293 | 9.44 |
| 9 | 11 | 123723 | 8.89 |
| 10 | 9 | 88810 | 10.13 |
| **Stratified by region** | | | |
| East midlands | 6 | 68071 | 8.81 |
| East of England | 5 | 80977 | 6.17 |
| London | 2 | 57222 | 3.50 |
| North East | 1 | 18318 | 5.46 |
| North West | 10 | 102327 | 9.77 |
| Northern Ireland | 1 | 11127 | 8.99 |
| Scotland | 2 | 18020 | 11.10 |
| South East | 8 | 115214 | 6.94 |
| South West | 4 | 64785 | 6.17 |
| Wales | 12 | 79507 | 15.09 |
| West Midlands | 12 | 70303 | 17.07 |
| Yorkshire and The Humber | 4 | 71248 | 5.61 |

*Definition 2: Probable PsA: psoriasis (PsO) plus arthritis diagnosis plus disease modifying anti-rheumatic drug (DMARD) treatment **Definition 3: Probable PsA: PsO plus arthritis diagnosis (Seronegative-Rheumatoid Arthritis, Axial Spondyloarthritis or axial arthritis).

| **Supplementary Table S9 - Observed incidence of PsA in CPRD in 2020** | | | |
| --- | --- | --- | --- |
| Stratification variable | Cases | Person time (years) | Incidence rate |
| **Case definition** | | | |
| All | 479 | 2843303 | 16.85 |
| Definition 1: Definite PsA | 375 | 2843303 | 13.19 |
| Definition 2: Probable PsA* | 72 | 2843303 | 2.53 |
| Definition 3: Probable PsA** | 32 | 2843303 | 1.13 |
| **Stratified by sex** | | | |
| Male | 259 | 1403237 | 15.68 |
| Female | 220 | 1440066 | 17.99 |
| **Stratified by age** | | | |
| 18-29 | 49 | 486398 | 10.07 |
| 30-49 | 192 | 956297 | 20.08 |
| 50-64 | 142 | 725243 | 19.58 |
| 65-79 | 83 | 492408 | 16.86 |
| 80+ | 13 | 182957 | 7.11 |
| **Stratified by IMD decile (1=most deprived, 10=least deprived).** | | | |
| 1 | 30 | 237989 | 12.61 |
| 2 | 55 | 265723 | 20.70 |
| 3 | 41 | 277371 | 14.78 |
| 4 | 35 | 213009 | 16.43 |
| 5 | 55 | 264862 | 20.77 |
| 6 | 38 | 275391 | 13.80 |
| 7 | 68 | 386712 | 17.58 |
| 8 | 39 | 259332 | 15.04 |
| 9 | 55 | 379979 | 14.47 |
| 10 | 63 | 282935 | 22.27 |
| **Stratified by region** | | | |
| East midlands*** |  | | |
| East of England | 2 | 12539 | 15.95 |
| London | 11 | 147736 | 7.45 |
| North East*** |  | | |
| North West | 23 | 164819 | 13.95 |
| Northern Ireland | 53 | 237512 | 22.31 |
| Scotland | 206 | 1155375 | 17.83 |
| South East | 35 | 211260 | 16.57 |
| South West | 6 | 37846 | 15.85 |
| Wales | 128 | 769159 | 16.64 |
| West Midlands | 14 | 101813 | 13.75 |
| Yorkshire and The Humber | 1 | 5244 | 19.07 |

*Definition 2: Probable PsA: psoriasis (PsO) plus arthritis diagnosis plus disease modifying anti-rheumatic drug (DMARD) treatment **Definition 3: Probable PsA: PsO plus arthritis diagnosis (Seronegative-Rheumatoid Arthritis, Axial Spondyloarthritis or axial arthritis). ***observed data not available for these regions in 2020.

# Estimated incidence between 1991 and 2020, all cases and stratified by case definition, sex, age, deprivation and region

| **Supplementary Table S10 – Annual estimates of PsA incidence in the UK between 1991 and 2020** | | | |
| --- | --- | --- | --- |
| Year | Incidence estimate | Lower confidence limit | Upper confidence limit |
| 1991 | 8.95 | 2.80 | 19.81 |
| 1992 | 8.76 | 2.74 | 19.29 |
| 1993 | 8.89 | 2.82 | 19.55 |
| 1994 | 9.34 | 2.97 | 20.47 |
| 1995 | 12.42 | 3.99 | 27.16 |
| 1996 | 10.74 | 3.45 | 23.40 |
| 1997 | 12.44 | 4.03 | 27.01 |
| 1998 | 13.61 | 4.46 | 29.42 |
| 1999 | 13.29 | 4.37 | 28.65 |
| 2000 | 16.04 | 5.29 | 34.59 |
| 2001 | 22.84 | 7.59 | 49.03 |
| 2002 | 23.15 | 7.72 | 49.60 |
| 2003 | 21.89 | 7.31 | 46.76 |
| 2004 | 21.72 | 7.26 | 46.42 |
| 2005 | 20.52 | 6.86 | 43.85 |
| 2006 | 20.64 | 6.92 | 44.06 |
| 2007 | 19.87 | 6.65 | 42.49 |
| 2008 | 20.13 | 6.74 | 43.03 |
| 2009 | 22.35 | 7.48 | 47.78 |
| 2010 | 20.96 | 7.01 | 44.76 |
| 2011 | 21.82 | 7.30 | 46.62 |
| 2012 | 22.26 | 7.45 | 47.54 |
| 2013 | 22.14 | 7.44 | 47.20 |
| 2014 | 21.15 | 7.08 | 45.22 |
| 2015 | 20.32 | 6.79 | 43.42 |
| 2016 | 19.57 | 6.53 | 41.95 |
| 2017 | 19.97 | 6.63 | 42.84 |
| 2018 | 21.38 | 7.12 | 45.79 |
| 2019 | 23.16 | 7.73 | 49.59 |
| 2020 | 15.08 | 5.02 | 32.32 |

| **Supplementary Table S11 – Annual estimates of PsA incidence in the UK between 1991 and 2020 stratified by sex** | | | | | | |
| --- | --- | --- | --- | --- | --- | --- |
| Year | Women | | | Men | | |
|  | Incidence estimate | Lower confidence limit | Upper confidence limit | Incidence estimate | Lower confidence limit | Upper confidence limit |
| 1991 | 9.47 | 2.98 | 20.80 | 8.44 | 2.66 | 18.53 |
| 1992 | 9.14 | 2.92 | 19.96 | 8.16 | 2.61 | 17.80 |
| 1993 | 9.35 | 3.02 | 20.23 | 8.34 | 2.69 | 18.04 |
| 1994 | 9.84 | 3.20 | 21.27 | 8.78 | 2.86 | 18.95 |
| 1995 | 13.05 | 4.27 | 28.25 | 11.64 | 3.81 | 25.19 |
| 1996 | 11.29 | 3.68 | 24.36 | 10.07 | 3.29 | 21.72 |
| 1997 | 13.00 | 4.29 | 28.01 | 11.59 | 3.83 | 24.96 |
| 1998 | 14.30 | 4.75 | 30.64 | 12.76 | 4.24 | 27.30 |
| 1999 | 13.96 | 4.66 | 29.81 | 12.45 | 4.16 | 26.58 |
| 2000 | 16.84 | 5.66 | 35.84 | 15.02 | 5.05 | 31.96 |
| 2001 | 23.94 | 8.08 | 50.76 | 21.35 | 7.21 | 45.26 |
| 2002 | 24.27 | 8.23 | 51.42 | 21.64 | 7.35 | 45.82 |
| 2003 | 23.01 | 7.82 | 48.65 | 20.52 | 6.98 | 43.33 |
| 2004 | 22.76 | 7.73 | 48.13 | 20.30 | 6.90 | 42.93 |
| 2005 | 21.47 | 7.32 | 45.34 | 19.15 | 6.53 | 40.42 |
| 2006 | 21.72 | 7.39 | 45.88 | 19.38 | 6.59 | 40.89 |
| 2007 | 20.86 | 7.10 | 44.08 | 18.60 | 6.33 | 39.28 |
| 2008 | 21.16 | 7.20 | 44.77 | 18.87 | 6.43 | 39.92 |
| 2009 | 23.49 | 8.01 | 49.55 | 20.95 | 7.15 | 44.18 |
| 2010 | 22.06 | 7.51 | 46.56 | 19.68 | 6.71 | 41.50 |
| 2011 | 22.89 | 7.81 | 48.24 | 20.41 | 6.97 | 42.99 |
| 2012 | 23.37 | 7.95 | 49.37 | 20.84 | 7.10 | 44.04 |
| 2013 | 23.23 | 7.93 | 48.94 | 20.72 | 7.08 | 43.65 |
| 2014 | 22.23 | 7.56 | 46.94 | 19.83 | 6.75 | 41.85 |
| 2015 | 21.37 | 7.27 | 45.13 | 19.06 | 6.49 | 40.23 |
| 2016 | 20.60 | 6.98 | 43.60 | 18.37 | 6.23 | 38.86 |
| 2017 | 20.87 | 7.07 | 44.20 | 18.62 | 6.31 | 39.40 |
| 2018 | 22.46 | 7.63 | 47.52 | 20.03 | 6.81 | 42.35 |
| 2019 | 24.21 | 8.19 | 51.25 | 21.59 | 7.31 | 45.69 |
| 2020 | 15.76 | 5.32 | 33.39 | 14.06 | 4.75 | 29.77 |

| **Supplementary Table S12 – Annual estimates of PsA incidence in the UK between 1991 and 2020 stratified by Age** | | | | | | | | | |
| --- | --- | --- | --- | --- | --- | --- | --- | --- | --- |
| Year | 18-29 | | | 30-49 | | | 50-64 | | |
|  | Incidence estimate | Lower confidence limit | Upper confidence limit | Incidence estimate | Lower confidence limit | Upper confidence limit | Incidence estimate | Lower confidence limit | Upper confidence limit |
| 1991 | 4.04 | 2.39 | 7.02 | 10.06 | 5.99 | 17.46 | 13.93 | 8.27 | 24.21 |
| 1992 | 3.95 | 2.34 | 6.84 | 9.84 | 5.87 | 17.01 | 13.63 | 8.10 | 23.64 |
| 1993 | 3.97 | 2.40 | 6.88 | 9.90 | 6.02 | 17.10 | 13.72 | 8.31 | 23.70 |
| 1994 | 4.18 | 2.55 | 7.21 | 10.43 | 6.39 | 17.92 | 14.45 | 8.84 | 24.87 |
| 1995 | 5.54 | 3.42 | 9.52 | 13.80 | 8.59 | 23.68 | 19.12 | 11.86 | 32.83 |
| 1996 | 4.81 | 2.97 | 8.25 | 11.99 | 7.46 | 20.53 | 16.62 | 10.32 | 28.47 |
| 1997 | 5.53 | 3.44 | 9.46 | 13.78 | 8.64 | 23.54 | 19.10 | 11.94 | 32.62 |
| 1998 | 6.10 | 3.83 | 10.43 | 15.19 | 9.62 | 25.92 | 21.05 | 13.27 | 35.93 |
| 1999 | 5.98 | 3.78 | 10.18 | 14.90 | 9.51 | 25.31 | 20.65 | 13.12 | 35.12 |
| 2000 | 7.22 | 4.59 | 12.28 | 17.98 | 11.54 | 30.53 | 24.91 | 15.94 | 42.33 |
| 2001 | 10.26 | 6.60 | 17.38 | 25.55 | 16.57 | 43.19 | 35.40 | 22.90 | 59.91 |
| 2002 | 10.37 | 6.69 | 17.55 | 25.84 | 16.76 | 43.67 | 35.81 | 23.16 | 60.50 |
| 2003 | 9.83 | 6.35 | 16.66 | 24.50 | 15.92 | 41.46 | 33.94 | 22.00 | 57.48 |
| 2004 | 9.75 | 6.29 | 16.50 | 24.28 | 15.78 | 41.03 | 33.64 | 21.82 | 56.88 |
| 2005 | 9.20 | 5.97 | 15.53 | 22.92 | 14.96 | 38.65 | 31.75 | 20.67 | 53.60 |
| 2006 | 9.28 | 6.02 | 15.67 | 23.11 | 15.12 | 38.99 | 32.02 | 20.88 | 54.03 |
| 2007 | 8.91 | 5.77 | 15.05 | 22.20 | 14.48 | 37.45 | 30.76 | 20.00 | 51.92 |
| 2008 | 9.05 | 5.86 | 15.28 | 22.54 | 14.70 | 38.00 | 31.22 | 20.30 | 52.66 |
| 2009 | 10.04 | 6.53 | 16.95 | 25.01 | 16.38 | 42.16 | 34.65 | 22.63 | 58.48 |
| 2010 | 9.45 | 6.14 | 15.98 | 23.55 | 15.39 | 39.71 | 32.63 | 21.27 | 55.06 |
| 2011 | 9.78 | 6.36 | 16.52 | 24.37 | 15.94 | 41.06 | 33.76 | 22.02 | 56.96 |
| 2012 | 9.99 | 6.49 | 16.86 | 24.89 | 16.27 | 41.98 | 34.49 | 22.47 | 58.17 |
| 2013 | 9.94 | 6.45 | 16.82 | 24.77 | 16.18 | 41.83 | 34.31 | 22.37 | 57.97 |
| 2014 | 9.50 | 6.15 | 16.06 | 23.66 | 15.43 | 39.96 | 32.78 | 21.33 | 55.33 |
| 2015 | 9.13 | 5.92 | 15.44 | 22.74 | 14.86 | 38.39 | 31.51 | 20.51 | 53.19 |
| 2016 | 8.80 | 5.67 | 14.90 | 21.92 | 14.22 | 37.03 | 30.37 | 19.64 | 51.33 |
| 2017 | 8.93 | 5.74 | 15.11 | 22.24 | 14.39 | 37.59 | 30.81 | 19.87 | 52.13 |
| 2018 | 9.60 | 6.18 | 16.28 | 23.91 | 15.49 | 40.48 | 33.12 | 21.41 | 56.10 |
| 2019 | 10.38 | 6.69 | 17.58 | 25.86 | 16.78 | 43.71 | 35.83 | 23.15 | 60.56 |
| 2020 | 6.77 | 4.36 | 11.46 | 16.87 | 10.93 | 28.53 | 23.37 | 15.10 | 39.56 |
|  |  |  |  |  |  |  |  |  |  |
| Year | 65-79 | | | 80+ | | |  |  |  |
|  | Incidence estimate | Lower confidence limit | Upper confidence limit | Incidence estimate | Lower confidence limit | Upper confidence limit |  |  |  |
| 1991 | 11.34 | 6.72 | 19.70 | 4.87 | 2.88 | 8.49 |  |  |  |
| 1992 | 11.09 | 6.59 | 19.25 | 4.77 | 2.82 | 8.27 |  |  |  |
| 1993 | 11.16 | 6.75 | 19.31 | 4.80 | 2.90 | 8.31 |  |  |  |
| 1994 | 11.76 | 7.19 | 20.26 | 5.05 | 3.08 | 8.71 |  |  |  |
| 1995 | 15.56 | 9.65 | 26.72 | 6.68 | 4.14 | 11.49 |  |  |  |
| 1996 | 13.52 | 8.38 | 23.17 | 5.81 | 3.59 | 9.97 |  |  |  |
| 1997 | 15.54 | 9.70 | 26.56 | 6.68 | 4.17 | 11.44 |  |  |  |
| 1998 | 17.13 | 10.78 | 29.27 | 7.36 | 4.62 | 12.58 |  |  |  |
| 1999 | 16.80 | 10.67 | 28.58 | 7.22 | 4.57 | 12.30 |  |  |  |
| 2000 | 20.27 | 12.95 | 34.47 | 8.71 | 5.55 | 14.83 |  |  |  |
| 2001 | 28.81 | 18.60 | 48.77 | 12.38 | 7.98 | 21.00 |  |  |  |
| 2002 | 29.14 | 18.83 | 49.31 | 12.52 | 8.07 | 21.23 |  |  |  |
| 2003 | 27.63 | 17.89 | 46.80 | 11.87 | 7.67 | 20.14 |  |  |  |
| 2004 | 27.38 | 17.74 | 46.33 | 11.76 | 7.61 | 19.93 |  |  |  |
| 2005 | 25.84 | 16.79 | 43.67 | 11.10 | 7.20 | 18.80 |  |  |  |
| 2006 | 26.06 | 16.97 | 44.03 | 11.20 | 7.28 | 18.95 |  |  |  |
| 2007 | 25.04 | 16.25 | 42.31 | 10.76 | 6.96 | 18.18 |  |  |  |
| 2008 | 25.41 | 16.51 | 42.90 | 10.92 | 7.08 | 18.47 |  |  |  |
| 2009 | 28.20 | 18.40 | 47.59 | 12.11 | 7.89 | 20.47 |  |  |  |
| 2010 | 26.56 | 17.28 | 44.83 | 11.41 | 7.41 | 19.31 |  |  |  |
| 2011 | 27.48 | 17.89 | 46.35 | 11.81 | 7.67 | 19.97 |  |  |  |
| 2012 | 28.07 | 18.26 | 47.39 | 12.06 | 7.83 | 20.40 |  |  |  |
| 2013 | 27.93 | 18.18 | 47.21 | 12.00 | 7.79 | 20.33 |  |  |  |
| 2014 | 26.68 | 17.33 | 45.06 | 11.46 | 7.43 | 19.39 |  |  |  |
| 2015 | 25.64 | 16.68 | 43.35 | 11.02 | 7.15 | 18.64 |  |  |  |
| 2016 | 24.72 | 15.97 | 41.75 | 10.62 | 6.85 | 17.98 |  |  |  |
| 2017 | 25.08 | 16.16 | 42.43 | 10.77 | 6.94 | 18.27 |  |  |  |
| 2018 | 26.96 | 17.40 | 45.70 | 11.58 | 7.46 | 19.65 |  |  |  |
| 2019 | 29.16 | 18.82 | 49.33 | 12.53 | 8.08 | 21.23 |  |  |  |
| 2020 | 19.02 | 12.28 | 32.20 | 8.17 | 5.25 | 13.88 |  |  |  |

| **Supplementary Table S13 – Annual estimates of PsA incidence in the UK between 1991 and 2020 stratified by IMD decile (1=most deprived, 10=least deprived).** | | | | | | | | | |
| --- | --- | --- | --- | --- | --- | --- | --- | --- | --- |
| Year | 1 (most deprived) | | | 2 | | | 3 | | |
|  | Incidence estimate | Lower confidence limit | Upper confidence limit | Incidence estimate | Lower confidence limit | Upper confidence limit | Incidence estimate | Lower confidence limit | Upper confidence limit |
| 1991 | 9.07 | 2.94 | 19.63 | 9.21 | 2.99 | 19.92 | 8.47 | 2.73 | 18.34 |
| 1992 | 8.85 | 2.89 | 19.22 | 8.99 | 2.94 | 19.52 | 8.27 | 2.69 | 17.99 |
| 1993 | 8.98 | 2.95 | 19.34 | 9.12 | 3.01 | 19.64 | 8.39 | 2.75 | 18.09 |
| 1994 | 9.50 | 3.13 | 20.39 | 9.65 | 3.18 | 20.70 | 8.87 | 2.91 | 19.11 |
| 1995 | 12.50 | 4.19 | 26.60 | 12.70 | 4.25 | 27.03 | 11.67 | 3.89 | 24.89 |
| 1996 | 10.88 | 3.66 | 23.14 | 11.06 | 3.73 | 23.49 | 10.16 | 3.41 | 21.66 |
| 1997 | 12.53 | 4.23 | 26.65 | 12.73 | 4.31 | 27.01 | 11.70 | 3.93 | 24.91 |
| 1998 | 13.76 | 4.69 | 29.06 | 13.98 | 4.76 | 29.49 | 12.85 | 4.35 | 27.19 |
| 1999 | 13.45 | 4.61 | 28.33 | 13.67 | 4.68 | 28.80 | 12.57 | 4.29 | 26.48 |
| 2000 | 16.22 | 5.57 | 34.01 | 16.47 | 5.67 | 34.61 | 15.14 | 5.18 | 31.85 |
| 2001 | 23.01 | 7.99 | 48.16 | 23.38 | 8.12 | 49.00 | 21.49 | 7.43 | 45.07 |
| 2002 | 23.33 | 8.11 | 48.76 | 23.70 | 8.24 | 49.65 | 21.79 | 7.55 | 45.60 |
| 2003 | 22.14 | 7.71 | 46.13 | 22.50 | 7.84 | 46.96 | 20.68 | 7.16 | 43.18 |
| 2004 | 21.97 | 7.65 | 45.93 | 22.32 | 7.77 | 46.61 | 20.51 | 7.11 | 42.92 |
| 2005 | 20.69 | 7.21 | 43.17 | 21.02 | 7.33 | 43.87 | 19.32 | 6.70 | 40.37 |
| 2006 | 20.94 | 7.31 | 43.69 | 21.27 | 7.42 | 44.39 | 19.55 | 6.79 | 40.82 |
| 2007 | 20.07 | 7.00 | 41.93 | 20.38 | 7.11 | 42.58 | 18.74 | 6.50 | 39.19 |
| 2008 | 20.38 | 7.11 | 42.47 | 20.70 | 7.23 | 43.19 | 19.03 | 6.61 | 39.78 |
| 2009 | 22.61 | 7.90 | 47.13 | 22.97 | 8.02 | 47.93 | 21.12 | 7.34 | 44.04 |
| 2010 | 21.20 | 7.40 | 44.14 | 21.54 | 7.52 | 44.98 | 19.80 | 6.88 | 41.34 |
| 2011 | 22.04 | 7.67 | 46.01 | 22.39 | 7.81 | 46.76 | 20.59 | 7.13 | 43.03 |
| 2012 | 22.50 | 7.85 | 46.94 | 22.86 | 7.98 | 47.74 | 21.02 | 7.30 | 43.93 |
| 2013 | 22.33 | 7.79 | 46.61 | 22.69 | 7.93 | 47.32 | 20.86 | 7.25 | 43.57 |
| 2014 | 21.36 | 7.45 | 44.51 | 21.70 | 7.58 | 45.24 | 19.95 | 6.92 | 41.65 |
| 2015 | 20.57 | 7.16 | 42.93 | 20.90 | 7.28 | 43.71 | 19.21 | 6.66 | 40.19 |
| 2016 | 19.80 | 6.88 | 41.44 | 20.11 | 6.99 | 42.11 | 18.49 | 6.40 | 38.75 |
| 2017 | 20.11 | 6.99 | 42.03 | 20.43 | 7.11 | 42.77 | 18.78 | 6.49 | 39.32 |
| 2018 | 21.64 | 7.51 | 45.30 | 21.99 | 7.64 | 45.98 | 20.21 | 6.97 | 42.38 |
| 2019 | 23.38 | 8.12 | 48.87 | 23.75 | 8.26 | 49.71 | 21.83 | 7.56 | 45.73 |
| 2020 | 15.21 | 5.27 | 31.85 | 15.45 | 5.36 | 32.36 | 14.20 | 4.90 | 29.74 |
|  |  |  |  |  |  |  |  |  |  |
| Year | 4 | | | 5 | | | 6 | | |
|  | Incidence estimate | Lower confidence limit | Upper confidence limit | Incidence estimate | Lower confidence limit | Upper confidence limit | Incidence estimate | Lower confidence limit | Upper confidence limit |
| 1991 | 8.39 | 2.71 | 18.15 | 8.65 | 2.79 | 18.78 | 8.90 | 2.87 | 19.32 |
| 1992 | 8.19 | 2.67 | 17.79 | 8.44 | 2.75 | 18.38 | 8.68 | 2.82 | 18.86 |
| 1993 | 8.31 | 2.73 | 17.90 | 8.56 | 2.80 | 18.48 | 8.81 | 2.89 | 18.99 |
| 1994 | 8.79 | 2.88 | 18.90 | 9.06 | 2.97 | 19.49 | 9.32 | 3.05 | 20.06 |
| 1995 | 11.56 | 3.86 | 24.60 | 11.92 | 3.97 | 25.41 | 12.26 | 4.08 | 26.15 |
| 1996 | 10.07 | 3.38 | 21.42 | 10.38 | 3.48 | 22.07 | 10.68 | 3.58 | 22.70 |
| 1997 | 11.60 | 3.91 | 24.63 | 11.95 | 4.02 | 25.44 | 12.29 | 4.13 | 26.17 |
| 1998 | 12.73 | 4.33 | 26.86 | 13.12 | 4.45 | 27.81 | 13.50 | 4.58 | 28.54 |
| 1999 | 12.45 | 4.26 | 26.20 | 12.83 | 4.38 | 27.10 | 13.20 | 4.50 | 27.81 |
| 2000 | 15.00 | 5.14 | 31.51 | 15.46 | 5.29 | 32.53 | 15.90 | 5.43 | 33.47 |
| 2001 | 21.29 | 7.37 | 44.60 | 21.94 | 7.58 | 46.10 | 22.57 | 7.79 | 47.37 |
| 2002 | 21.59 | 7.48 | 45.18 | 22.25 | 7.69 | 46.56 | 22.88 | 7.91 | 48.02 |
| 2003 | 20.49 | 7.13 | 42.76 | 21.12 | 7.32 | 44.15 | 21.72 | 7.53 | 45.43 |
| 2004 | 20.32 | 7.06 | 42.45 | 20.95 | 7.26 | 43.79 | 21.55 | 7.46 | 45.02 |
| 2005 | 19.15 | 6.65 | 39.98 | 19.73 | 6.84 | 41.25 | 20.30 | 7.03 | 42.47 |
| 2006 | 19.37 | 6.74 | 40.47 | 19.97 | 6.93 | 41.80 | 20.53 | 7.12 | 42.97 |
| 2007 | 18.57 | 6.46 | 38.76 | 19.13 | 6.64 | 39.99 | 19.68 | 6.83 | 41.16 |
| 2008 | 18.85 | 6.57 | 39.33 | 19.43 | 6.75 | 40.63 | 19.99 | 6.94 | 41.77 |
| 2009 | 20.92 | 7.28 | 43.63 | 21.56 | 7.49 | 45.04 | 22.18 | 7.69 | 46.32 |
| 2010 | 19.62 | 6.83 | 40.87 | 20.22 | 7.02 | 42.24 | 20.80 | 7.23 | 43.44 |
| 2011 | 20.40 | 7.08 | 42.63 | 21.02 | 7.28 | 43.90 | 21.62 | 7.49 | 45.21 |
| 2012 | 20.82 | 7.24 | 43.46 | 21.46 | 7.44 | 44.91 | 22.07 | 7.65 | 46.16 |
| 2013 | 20.66 | 7.19 | 43.07 | 21.30 | 7.40 | 44.51 | 21.91 | 7.60 | 45.80 |
| 2014 | 19.76 | 6.88 | 41.28 | 20.37 | 7.07 | 42.57 | 20.95 | 7.28 | 43.76 |
| 2015 | 19.03 | 6.61 | 39.81 | 19.62 | 6.79 | 41.06 | 20.18 | 6.98 | 42.25 |
| 2016 | 18.32 | 6.35 | 38.35 | 18.88 | 6.52 | 39.60 | 19.42 | 6.71 | 40.63 |
| 2017 | 18.60 | 6.44 | 38.91 | 19.17 | 6.63 | 40.25 | 19.72 | 6.82 | 41.30 |
| 2018 | 20.02 | 6.92 | 41.90 | 20.64 | 7.11 | 43.32 | 21.23 | 7.32 | 44.52 |
| 2019 | 21.63 | 7.51 | 45.26 | 22.29 | 7.71 | 46.64 | 22.93 | 7.93 | 48.14 |
| 2020 | 14.07 | 4.87 | 29.50 | 14.50 | 5.00 | 30.44 | 14.92 | 5.14 | 31.35 |
|  |  |  |  |  |  |  |  |  |  |
|  |  |  |  |  |  |  |  |  |  |
| Year | 7 | | | 8 | | | 9 | | |
|  | Incidence estimate | Lower confidence limit | Upper confidence limit | Incidence estimate | Lower confidence limit | Upper confidence limit | Incidence estimate | Lower confidence limit | Upper confidence limit |
| 1991 | 9.29 | 2.99 | 20.18 | 8.63 | 2.79 | 18.77 | 9.37 | 3.03 | 20.39 |
| 1992 | 9.07 | 2.94 | 19.78 | 8.43 | 2.74 | 18.29 | 9.15 | 2.98 | 19.91 |
| 1993 | 9.21 | 3.01 | 19.87 | 8.55 | 2.80 | 18.42 | 9.28 | 3.04 | 20.03 |
| 1994 | 9.74 | 3.18 | 20.99 | 9.04 | 2.96 | 19.45 | 9.82 | 3.22 | 21.14 |
| 1995 | 12.81 | 4.26 | 27.32 | 11.90 | 3.96 | 25.39 | 12.92 | 4.32 | 27.58 |
| 1996 | 11.16 | 3.73 | 23.82 | 10.36 | 3.47 | 22.07 | 11.25 | 3.77 | 23.99 |
| 1997 | 12.85 | 4.30 | 27.38 | 11.93 | 4.01 | 25.38 | 12.96 | 4.36 | 27.63 |
| 1998 | 14.10 | 4.76 | 29.95 | 13.10 | 4.44 | 27.71 | 14.22 | 4.82 | 30.12 |
| 1999 | 13.79 | 4.70 | 29.17 | 12.81 | 4.37 | 27.02 | 13.91 | 4.75 | 29.35 |
| 2000 | 16.62 | 5.67 | 34.95 | 15.44 | 5.27 | 32.50 | 16.76 | 5.74 | 35.31 |
| 2001 | 23.59 | 8.11 | 49.61 | 21.91 | 7.58 | 45.98 | 23.79 | 8.23 | 49.96 |
| 2002 | 23.91 | 8.24 | 50.24 | 22.21 | 7.68 | 46.52 | 24.12 | 8.35 | 50.60 |
| 2003 | 22.69 | 7.84 | 47.55 | 21.08 | 7.31 | 44.08 | 22.89 | 7.95 | 47.87 |
| 2004 | 22.52 | 7.78 | 47.12 | 20.91 | 7.25 | 43.67 | 22.71 | 7.88 | 47.49 |
| 2005 | 21.21 | 7.33 | 44.42 | 19.70 | 6.83 | 41.17 | 21.39 | 7.42 | 44.78 |
| 2006 | 21.46 | 7.41 | 44.99 | 19.93 | 6.92 | 41.69 | 21.64 | 7.52 | 45.29 |
| 2007 | 20.57 | 7.11 | 43.17 | 19.10 | 6.63 | 39.98 | 20.74 | 7.20 | 43.48 |
| 2008 | 20.88 | 7.24 | 43.77 | 19.40 | 6.75 | 40.51 | 21.06 | 7.32 | 44.08 |
| 2009 | 23.18 | 8.02 | 48.45 | 21.53 | 7.48 | 45.02 | 23.37 | 8.12 | 48.86 |
| 2010 | 21.73 | 7.53 | 45.45 | 20.18 | 7.01 | 42.07 | 21.92 | 7.62 | 45.80 |
| 2011 | 22.59 | 7.81 | 47.31 | 20.98 | 7.27 | 43.90 | 22.79 | 7.90 | 47.74 |
| 2012 | 23.06 | 7.98 | 48.26 | 21.42 | 7.44 | 44.81 | 23.26 | 8.08 | 48.73 |
| 2013 | 22.89 | 7.92 | 47.88 | 21.26 | 7.39 | 44.42 | 23.09 | 8.02 | 48.26 |
| 2014 | 21.89 | 7.59 | 45.80 | 20.33 | 7.07 | 42.49 | 22.08 | 7.68 | 46.13 |
| 2015 | 21.08 | 7.28 | 44.21 | 19.58 | 6.79 | 40.99 | 21.26 | 7.37 | 44.52 |
| 2016 | 20.29 | 6.99 | 42.57 | 18.85 | 6.52 | 39.49 | 20.47 | 7.08 | 42.96 |
| 2017 | 20.61 | 7.10 | 43.20 | 19.14 | 6.63 | 40.08 | 20.78 | 7.20 | 43.60 |
| 2018 | 22.18 | 7.63 | 46.62 | 20.60 | 7.12 | 43.18 | 22.37 | 7.73 | 46.98 |
| 2019 | 23.96 | 8.25 | 50.30 | 22.25 | 7.70 | 46.58 | 24.16 | 8.37 | 50.65 |
| 2020 | 15.59 | 5.36 | 32.73 | 14.48 | 5.00 | 30.39 | 15.72 | 5.42 | 33.09 |
|  |  |  |  |  |  |  |  |  |  |
|  |  |  |  |  |  |  |  |  |  |
| Year | 10 (least deprived) | | |  |  |  |  |  |  |
|  | Incidence estimate | Lower confidence limit | Upper confidence limit |  |  |  |  |  |  |
| 1991 | 8.75 | 2.83 | 19.01 |  |  |  |  |  |  |
| 1992 | 8.54 | 2.78 | 18.56 |  |  |  |  |  |  |
| 1993 | 8.66 | 2.85 | 18.72 |  |  |  |  |  |  |
| 1994 | 9.16 | 3.01 | 19.70 |  |  |  |  |  |  |
| 1995 | 12.06 | 4.03 | 25.71 |  |  |  |  |  |  |
| 1996 | 10.50 | 3.52 | 22.34 |  |  |  |  |  |  |
| 1997 | 12.09 | 4.07 | 25.79 |  |  |  |  |  |  |
| 1998 | 13.27 | 4.51 | 28.07 |  |  |  |  |  |  |
| 1999 | 12.98 | 4.44 | 27.35 |  |  |  |  |  |  |
| 2000 | 15.64 | 5.37 | 32.91 |  |  |  |  |  |  |
| 2001 | 22.20 | 7.68 | 46.61 |  |  |  |  |  |  |
| 2002 | 22.51 | 7.79 | 47.18 |  |  |  |  |  |  |
| 2003 | 21.36 | 7.42 | 44.61 |  |  |  |  |  |  |
| 2004 | 21.19 | 7.36 | 44.39 |  |  |  |  |  |  |
| 2005 | 19.96 | 6.93 | 41.67 |  |  |  |  |  |  |
| 2006 | 20.20 | 7.02 | 42.21 |  |  |  |  |  |  |
| 2007 | 19.36 | 6.72 | 40.53 |  |  |  |  |  |  |
| 2008 | 19.66 | 6.85 | 41.08 |  |  |  |  |  |  |
| 2009 | 21.82 | 7.58 | 45.56 |  |  |  |  |  |  |
| 2010 | 20.45 | 7.12 | 42.71 |  |  |  |  |  |  |
| 2011 | 21.27 | 7.38 | 44.46 |  |  |  |  |  |  |
| 2012 | 21.71 | 7.54 | 45.38 |  |  |  |  |  |  |
| 2013 | 21.55 | 7.51 | 44.99 |  |  |  |  |  |  |
| 2014 | 20.61 | 7.18 | 43.04 |  |  |  |  |  |  |
| 2015 | 19.84 | 6.89 | 41.51 |  |  |  |  |  |  |
| 2016 | 19.10 | 6.61 | 39.99 |  |  |  |  |  |  |
| 2017 | 19.40 | 6.72 | 40.66 |  |  |  |  |  |  |
| 2018 | 20.88 | 7.22 | 43.75 |  |  |  |  |  |  |
| 2019 | 22.55 | 7.82 | 47.23 |  |  |  |  |  |  |
| 2020 | 14.67 | 5.07 | 30.83 |  |  |  |  |  |  |

| **Supplementary Table S14 – Annual estimates of PsA incidence in the UK between 1991 and 2020 stratified by Region** | | | | | | | | | |
| --- | --- | --- | --- | --- | --- | --- | --- | --- | --- |
| Year | East midlands | | | East of England | | | London | | |
|  | Incidence estimate | Lower confidence limit | Upper confidence limit | Incidence estimate | Lower confidence limit | Upper confidence limit | Incidence estimate | Lower confidence limit | Upper confidence limit |
| 1991 | 8.05 | 2.81 | 15.77 | 7.71 | 2.70 | 15.03 | 6.46 | 2.25 | 12.69 |
| 1992 | 7.84 | 2.75 | 15.27 | 7.51 | 2.65 | 14.58 | 6.30 | 2.21 | 12.29 |
| 1993 | 7.95 | 2.86 | 15.26 | 7.61 | 2.75 | 14.56 | 6.39 | 2.29 | 12.30 |
| 1994 | 8.40 | 3.00 | 16.13 | 8.05 | 2.88 | 15.41 | 6.75 | 2.39 | 12.99 |
| 1995 | 11.04 | 4.01 | 20.92 | 10.57 | 3.86 | 19.91 | 8.87 | 3.21 | 16.80 |
| 1996 | 9.65 | 3.50 | 18.40 | 9.24 | 3.37 | 17.49 | 7.74 | 2.81 | 14.74 |
| 1997 | 11.06 | 4.06 | 20.89 | 10.59 | 3.90 | 19.90 | 8.88 | 3.25 | 16.74 |
| 1998 | 12.19 | 4.53 | 22.82 | 11.67 | 4.35 | 21.77 | 9.79 | 3.62 | 18.34 |
| 1999 | 11.94 | 4.46 | 22.24 | 11.43 | 4.28 | 21.22 | 9.58 | 3.58 | 17.87 |
| 2000 | 14.39 | 5.35 | 26.76 | 13.78 | 5.17 | 25.46 | 11.55 | 4.30 | 21.48 |
| 2001 | 20.43 | 7.68 | 37.80 | 19.56 | 7.40 | 36.05 | 16.40 | 6.16 | 30.37 |
| 2002 | 20.71 | 7.82 | 38.19 | 19.83 | 7.54 | 36.40 | 16.63 | 6.28 | 30.69 |
| 2003 | 19.64 | 7.43 | 36.12 | 18.80 | 7.16 | 34.48 | 15.77 | 5.96 | 29.05 |
| 2004 | 19.47 | 7.39 | 35.82 | 18.64 | 7.12 | 34.13 | 15.63 | 5.92 | 28.78 |
| 2005 | 18.36 | 6.98 | 33.64 | 17.58 | 6.73 | 32.04 | 14.74 | 5.60 | 27.03 |
| 2006 | 18.55 | 7.04 | 34.06 | 17.76 | 6.78 | 32.44 | 14.89 | 5.65 | 27.38 |
| 2007 | 17.82 | 6.78 | 32.68 | 17.06 | 6.53 | 31.18 | 14.31 | 5.44 | 26.30 |
| 2008 | 18.12 | 6.88 | 33.31 | 17.35 | 6.63 | 31.73 | 14.54 | 5.53 | 26.75 |
| 2009 | 20.07 | 7.64 | 36.78 | 19.22 | 7.36 | 35.09 | 16.11 | 6.13 | 29.58 |
| 2010 | 18.85 | 7.14 | 34.64 | 18.04 | 6.88 | 32.96 | 15.13 | 5.74 | 27.77 |
| 2011 | 19.50 | 7.43 | 35.69 | 18.67 | 7.14 | 34.02 | 15.65 | 5.94 | 28.69 |
| 2012 | 19.94 | 7.56 | 36.60 | 19.09 | 7.28 | 34.91 | 16.01 | 6.07 | 29.42 |
| 2013 | 19.86 | 7.55 | 36.47 | 19.01 | 7.27 | 34.73 | 15.94 | 6.06 | 29.26 |
| 2014 | 18.96 | 7.19 | 34.81 | 18.15 | 6.92 | 33.20 | 15.22 | 5.78 | 27.97 |
| 2015 | 18.25 | 6.92 | 33.57 | 17.47 | 6.67 | 31.94 | 14.65 | 5.55 | 26.96 |
| 2016 | 17.53 | 6.63 | 32.29 | 16.78 | 6.39 | 30.75 | 14.07 | 5.33 | 25.93 |
| 2017 | 17.87 | 6.74 | 32.98 | 17.10 | 6.49 | 31.47 | 14.34 | 5.40 | 26.50 |
| 2018 | 19.17 | 7.27 | 35.24 | 18.35 | 7.00 | 33.55 | 15.39 | 5.83 | 28.29 |
| 2019 | 20.68 | 7.83 | 38.10 | 19.79 | 7.56 | 36.30 | 16.59 | 6.30 | 30.62 |
| 2020 | 13.51 | 5.08 | 24.95 | 12.93 | 4.90 | 23.81 | 10.84 | 4.09 | 20.09 |
| Year | North East | | | North West | | | Northern Ireland | | |
|  | Incidence estimate | Lower confidence limit | Upper confidence limit | Incidence estimate | Lower confidence limit | Upper confidence limit | Incidence estimate | Lower confidence limit | Upper confidence limit |
| 1991 | 9.29 | 3.24 | 18.19 | 9.66 | 3.39 | 18.87 | 13.95 | 4.96 | 26.92 |
| 1992 | 9.06 | 3.17 | 17.73 | 9.41 | 3.32 | 18.27 | 13.59 | 4.85 | 26.16 |
| 1993 | 9.18 | 3.28 | 17.66 | 9.54 | 3.45 | 18.27 | 13.79 | 5.04 | 26.11 |
| 1994 | 9.70 | 3.47 | 18.62 | 10.08 | 3.61 | 19.30 | 14.57 | 5.28 | 27.62 |
| 1995 | 12.74 | 4.62 | 24.06 | 13.25 | 4.84 | 24.93 | 19.14 | 7.08 | 35.67 |
| 1996 | 11.14 | 4.05 | 21.24 | 11.58 | 4.24 | 21.90 | 16.72 | 6.21 | 31.37 |
| 1997 | 12.77 | 4.67 | 24.17 | 13.28 | 4.90 | 24.93 | 19.17 | 7.18 | 35.57 |
| 1998 | 14.08 | 5.21 | 26.36 | 14.63 | 5.46 | 27.29 | 21.14 | 8.01 | 38.95 |
| 1999 | 13.78 | 5.12 | 25.78 | 14.33 | 5.39 | 26.59 | 20.69 | 7.89 | 37.95 |
| 2000 | 16.61 | 6.20 | 30.93 | 17.27 | 6.48 | 31.93 | 24.95 | 9.52 | 45.63 |
| 2001 | 23.59 | 8.87 | 43.70 | 24.52 | 9.30 | 45.08 | 35.41 | 13.61 | 64.40 |
| 2002 | 23.91 | 9.02 | 44.23 | 24.85 | 9.47 | 45.57 | 35.90 | 13.85 | 65.16 |
| 2003 | 22.67 | 8.58 | 41.84 | 23.57 | 8.99 | 43.17 | 34.05 | 13.17 | 61.69 |
| 2004 | 22.48 | 8.51 | 41.38 | 23.37 | 8.93 | 42.75 | 33.76 | 13.08 | 61.10 |
| 2005 | 21.20 | 8.06 | 38.91 | 22.03 | 8.45 | 40.13 | 31.83 | 12.37 | 57.35 |
| 2006 | 21.42 | 8.11 | 39.46 | 22.26 | 8.52 | 40.60 | 32.16 | 12.47 | 58.09 |
| 2007 | 20.58 | 7.81 | 37.84 | 21.39 | 8.21 | 39.01 | 30.89 | 12.02 | 55.74 |
| 2008 | 20.92 | 7.93 | 38.52 | 21.74 | 8.33 | 39.72 | 31.41 | 12.19 | 56.80 |
| 2009 | 23.18 | 8.81 | 42.62 | 24.09 | 9.24 | 43.92 | 34.80 | 13.51 | 62.82 |
| 2010 | 21.75 | 8.27 | 39.99 | 22.61 | 8.66 | 41.26 | 32.67 | 12.67 | 58.96 |
| 2011 | 22.51 | 8.55 | 41.28 | 23.40 | 8.98 | 42.62 | 33.80 | 13.14 | 60.94 |
| 2012 | 23.02 | 8.70 | 42.43 | 23.93 | 9.14 | 43.71 | 34.56 | 13.41 | 62.56 |
| 2013 | 22.92 | 8.69 | 42.15 | 23.83 | 9.14 | 43.48 | 34.42 | 13.38 | 62.16 |
| 2014 | 21.89 | 8.29 | 40.36 | 22.75 | 8.73 | 41.56 | 32.87 | 12.75 | 59.41 |
| 2015 | 21.07 | 7.97 | 38.87 | 21.90 | 8.38 | 40.03 | 31.64 | 12.26 | 57.24 |
| 2016 | 20.24 | 7.65 | 37.33 | 21.03 | 8.02 | 38.51 | 30.38 | 11.76 | 55.09 |
| 2017 | 20.63 | 7.75 | 38.18 | 21.44 | 8.15 | 39.37 | 30.97 | 11.93 | 56.22 |
| 2018 | 22.13 | 8.38 | 40.84 | 23.00 | 8.80 | 42.13 | 33.23 | 12.88 | 60.08 |
| 2019 | 23.87 | 9.03 | 44.20 | 24.81 | 9.49 | 45.48 | 35.83 | 13.89 | 64.93 |
| 2020 | 15.60 | 5.87 | 28.87 | 16.21 | 6.16 | 29.82 | 23.41 | 9.03 | 42.57 |
|  |  |  |  |  |  |  |  |  |  |
|  |  |  |  |  |  |  |  |  |  |
| Year | Scotland | | | South East | | | South West | | |
|  | Incidence estimate | Lower confidence limit | Upper confidence limit | Incidence estimate | Lower confidence limit | Upper confidence limit | Incidence estimate | Lower confidence limit | Upper confidence limit |
| 1991 | 8.74 | 3.11 | 16.82 | 8.01 | 2.83 | 15.53 | 8.82 | 3.10 | 17.14 |
| 1992 | 8.51 | 3.05 | 16.29 | 7.81 | 2.78 | 15.05 | 8.59 | 3.04 | 16.62 |
| 1993 | 8.63 | 3.17 | 16.31 | 7.92 | 2.88 | 15.06 | 8.71 | 3.16 | 16.60 |
| 1994 | 9.12 | 3.32 | 17.22 | 8.37 | 3.02 | 15.89 | 9.21 | 3.31 | 17.53 |
| 1995 | 11.99 | 4.46 | 22.27 | 10.99 | 4.05 | 20.56 | 12.10 | 4.44 | 22.69 |
| 1996 | 10.47 | 3.90 | 19.55 | 9.60 | 3.54 | 18.07 | 10.57 | 3.88 | 19.93 |
| 1997 | 12.01 | 4.51 | 22.22 | 11.01 | 4.10 | 20.53 | 12.12 | 4.49 | 22.68 |
| 1998 | 13.24 | 5.04 | 24.30 | 12.14 | 4.56 | 22.48 | 13.36 | 5.00 | 24.80 |
| 1999 | 12.96 | 4.96 | 23.71 | 11.88 | 4.50 | 21.89 | 13.08 | 4.94 | 24.13 |
| 2000 | 15.62 | 5.98 | 28.49 | 14.33 | 5.42 | 26.34 | 15.77 | 5.94 | 29.03 |
| 2001 | 22.18 | 8.57 | 40.21 | 20.34 | 7.76 | 37.18 | 22.38 | 8.52 | 40.94 |
| 2002 | 22.48 | 8.73 | 40.63 | 20.62 | 7.93 | 37.56 | 22.69 | 8.68 | 41.43 |
| 2003 | 21.32 | 8.29 | 38.49 | 19.55 | 7.52 | 35.57 | 21.52 | 8.24 | 39.27 |
| 2004 | 21.14 | 8.24 | 38.09 | 19.38 | 7.47 | 35.25 | 21.33 | 8.19 | 38.87 |
| 2005 | 19.93 | 7.79 | 35.80 | 18.28 | 7.07 | 33.10 | 20.12 | 7.73 | 36.50 |
| 2006 | 20.14 | 7.86 | 36.23 | 18.47 | 7.13 | 33.48 | 20.32 | 7.80 | 36.96 |
| 2007 | 19.35 | 7.56 | 34.79 | 17.74 | 6.87 | 32.17 | 19.52 | 7.51 | 35.50 |
| 2008 | 19.67 | 7.67 | 35.43 | 18.04 | 6.97 | 32.74 | 19.85 | 7.63 | 36.15 |
| 2009 | 21.79 | 8.51 | 39.18 | 19.98 | 7.73 | 36.20 | 21.99 | 8.46 | 39.98 |
| 2010 | 20.46 | 7.97 | 36.80 | 18.76 | 7.23 | 34.04 | 20.64 | 7.92 | 37.53 |
| 2011 | 21.17 | 8.27 | 38.00 | 19.41 | 7.51 | 35.15 | 21.36 | 8.22 | 38.78 |
| 2012 | 21.65 | 8.43 | 39.03 | 19.85 | 7.65 | 36.04 | 21.84 | 8.38 | 39.72 |
| 2013 | 21.56 | 8.43 | 38.75 | 19.77 | 7.65 | 35.86 | 21.75 | 8.38 | 39.53 |
| 2014 | 20.58 | 8.03 | 37.09 | 18.87 | 7.29 | 34.30 | 20.77 | 7.99 | 37.83 |
| 2015 | 19.81 | 7.73 | 35.72 | 18.17 | 7.01 | 33.01 | 20.00 | 7.67 | 36.38 |
| 2016 | 19.03 | 7.40 | 34.28 | 17.45 | 6.71 | 31.75 | 19.20 | 7.35 | 34.99 |
| 2017 | 19.39 | 7.51 | 35.14 | 17.78 | 6.82 | 32.46 | 19.57 | 7.47 | 35.78 |
| 2018 | 20.81 | 8.11 | 37.48 | 19.08 | 7.35 | 34.69 | 21.00 | 8.06 | 38.25 |
| 2019 | 22.44 | 8.75 | 40.54 | 20.58 | 7.94 | 37.47 | 22.65 | 8.69 | 41.36 |
| 2020 | 14.66 | 5.69 | 26.56 | 13.45 | 5.15 | 24.60 | 14.80 | 5.65 | 27.11 |
| Year | Wales | | | West Midlands | | | Yorkshire and The Humber | | |
|  | Incidence estimate | Lower confidence limit | Upper confidence limit | Incidence estimate | Lower confidence limit | Upper confidence limit | Incidence estimate | Lower confidence limit | Upper confidence limit |
| 1991 | 8.95 | 3.20 | 17.20 | 7.97 | 2.80 | 15.51 | 8.58 | 2.99 | 16.86 |
| 1992 | 8.72 | 3.13 | 16.69 | 7.77 | 2.75 | 15.03 | 8.36 | 2.91 | 16.35 |
| 1993 | 8.84 | 3.25 | 16.67 | 7.88 | 2.85 | 15.03 | 8.47 | 3.04 | 16.35 |
| 1994 | 9.34 | 3.41 | 17.58 | 8.33 | 2.98 | 15.91 | 8.95 | 3.18 | 17.30 |
| 1995 | 12.27 | 4.58 | 22.75 | 10.94 | 4.01 | 20.53 | 11.77 | 4.26 | 22.38 |
| 1996 | 10.72 | 4.01 | 19.99 | 9.56 | 3.52 | 18.07 | 10.28 | 3.72 | 19.57 |
| 1997 | 12.30 | 4.64 | 22.68 | 10.96 | 4.06 | 20.53 | 11.79 | 4.31 | 22.37 |
| 1998 | 13.56 | 5.17 | 24.85 | 12.08 | 4.52 | 22.46 | 12.99 | 4.79 | 24.44 |
| 1999 | 13.27 | 5.10 | 24.20 | 11.82 | 4.46 | 21.86 | 12.72 | 4.73 | 23.80 |
| 2000 | 16.00 | 6.14 | 29.06 | 14.26 | 5.37 | 26.27 | 15.33 | 5.69 | 28.58 |
| 2001 | 22.71 | 8.80 | 41.04 | 20.24 | 7.70 | 37.09 | 21.77 | 8.16 | 40.44 |
| 2002 | 23.02 | 8.97 | 41.53 | 20.51 | 7.84 | 37.54 | 22.06 | 8.31 | 40.90 |
| 2003 | 21.84 | 8.51 | 39.32 | 19.45 | 7.44 | 35.55 | 20.92 | 7.89 | 38.75 |
| 2004 | 21.65 | 8.46 | 38.92 | 19.29 | 7.40 | 35.19 | 20.74 | 7.85 | 38.31 |
| 2005 | 20.41 | 8.00 | 36.59 | 18.19 | 7.00 | 33.05 | 19.56 | 7.41 | 36.01 |
| 2006 | 20.62 | 8.06 | 37.04 | 18.37 | 7.06 | 33.49 | 19.76 | 7.49 | 36.43 |
| 2007 | 19.82 | 7.76 | 35.58 | 17.65 | 6.80 | 32.14 | 18.99 | 7.19 | 34.99 |
| 2008 | 20.14 | 7.87 | 36.22 | 17.95 | 6.90 | 32.70 | 19.30 | 7.30 | 35.68 |
| 2009 | 22.32 | 8.74 | 40.04 | 19.88 | 7.65 | 36.17 | 21.39 | 8.10 | 39.41 |
| 2010 | 20.95 | 8.19 | 37.63 | 18.67 | 7.16 | 34.00 | 20.07 | 7.58 | 37.04 |
| 2011 | 21.68 | 8.49 | 38.85 | 19.32 | 7.43 | 35.10 | 20.77 | 7.88 | 38.26 |
| 2012 | 22.17 | 8.66 | 39.80 | 19.75 | 7.58 | 35.99 | 21.24 | 8.02 | 39.26 |
| 2013 | 22.07 | 8.66 | 39.63 | 19.67 | 7.57 | 35.80 | 21.15 | 8.03 | 38.95 |
| 2014 | 21.08 | 8.24 | 37.87 | 18.78 | 7.22 | 34.24 | 20.20 | 7.65 | 37.27 |
| 2015 | 20.29 | 7.93 | 36.48 | 18.08 | 6.93 | 32.97 | 19.44 | 7.34 | 35.90 |
| 2016 | 19.49 | 7.59 | 35.09 | 17.36 | 6.64 | 31.72 | 18.67 | 7.03 | 34.53 |
| 2017 | 19.86 | 7.72 | 35.84 | 17.70 | 6.75 | 32.39 | 19.03 | 7.15 | 35.27 |
| 2018 | 21.31 | 8.34 | 38.32 | 18.99 | 7.28 | 34.66 | 20.42 | 7.71 | 37.74 |
| 2019 | 22.98 | 8.98 | 41.39 | 20.48 | 7.86 | 37.44 | 22.02 | 8.31 | 40.77 |
| 2020 | 15.02 | 5.84 | 27.11 | 13.38 | 5.10 | 24.54 | 14.39 | 5.41 | 26.73 |
